# Structure–stability relationships of pure cannabinoids and their cocrystals under forced degradation conditions

**DOI:** 10.1186/s42238-026-00474-0

**Published:** 2026-07-21

**Authors:** Adéla Koryťáková, Argyro Chatziadi, Jakub Heřt, Jan Rohlíček, Eliška Zmeškalová, Josef Beránek, Miroslav Šoóš

**Affiliations:** 1 Department of Chemical Engineering, University of Chemistry and Technology in Prague, Technická 3, Prague 6, 16628 Czech Republic; 2 Institute of Physics of the Czech Academy of Sciences, Na Slovance 2, Prague 8, 182 00 Czech Republic; 3 Zentiva k.s., U Kabelovny 130, Prague 10, 10237 Czech Republic

**Keywords:** Cannabigerol, Cannabidiol, Cannabinol, Cocrystals, Forced degradation

## Abstract

Cannabinoids have attracted increasing scientific interest due to their diverse activities and therapeutic potential. However, cannabinoids suffer from limited physicochemical properties, including low melting point, low aqueous solubility, and stability issues. Cocrystal formation offers a promising approach to overcome these limitations. In this study, we investigate the solid-state stability of cannabinoids, namely cannabidiol (CBD), cannabinol (CBN), and cannabigerol (CBG), and their cocrystals under thermal, pH-dependent, oxidative, and photolytic degradation stress conditions, with a focus on exploring the structure-stability relationships. The results revealed distinct stability trends. We observed that 4,4’-bipyridine improved the thermal stability compared to the pure cannabinoids, highlighting the role of coformer selection. pH-dependent studies generally showed higher degradation rates at intermediate pH values, whereas photostability experiments showed negligible instability in most samples. Oxidative stressing indicated that radical-mediated oxidation had a stronger impact on stability than peroxide-mediated oxidation. Overall, the results indicate that coformer choice and surface exposure of heteroatoms are key factors influencing the stability of cannabinoid cocrystals.

**Supplementary Information:**

The online version contains supplementary material available at https://doi.org/10.1186/s42238-026-00474-0.

## Introduction

Cannabinoids, a broad group of natural compounds derived from the *Cannabis sativa* plant, have attracted considerable scientific and pharmaceutical interest due to their diverse biological activities and therapeutic potential. Among the major non-psychoactive cannabinoids are cannabidiol (CBD), cannabinol (CBN), and cannabigerol (CBG), which represent structurally related molecules with distinct chemical and biological profiles (Ryšánek et al. 2025; Dawidowicz et al. 2021; Khouchlaa et al. 2024; Wong and Cairns 2019; Zagožen et al. 2021). CBD is the most extensively studied cannabinoid and has led to the approval of cannabinoid-based medicine such as Sativex (2026) and Epidiolex (2026). In comparison, CBN has been reported to exhibit anti-inflammatory and analgesic effects (Wong and Cairns 2019; Zaiachuk et al. 2023), while CBG, often referred as a precursor of other cannabinoids (Tahir et al. 2021), has demonstrated antimicrobial, anticancer, and appetite-enhancing effects (Anokwuru et al. 2022; Calapai et al. 2022).

Despite their promising pharmacological activities, the pharmaceutical development of cannabinoids is challenged by unfavourable physicochemical properties, including low melting points (Koryt’áková et al. 2025; Koryťáková et al. 2025; Zmeškalová et al. 2025), poor aqueous solubility (Khouchlaa et al. 2024; Millar et al. 2018) and limited chemical stability (Fraguas-Sanchez et al. 2020; Jaidee et al. 2021). Various formulations and solid-state strategies have therefore been explored to overcome these limitations (Hu et al. 2004; Khan et al. 2022; Hriňová et al. 2022; Aitipamula et al. 2012; Vinarov et al. 2018; Alizadeh et al. 2018). Among them, the formation of cocrystals has emerged as an effective approach to modify key physicochemical properties, such as solubility and thermal stability, without chemically altering the Active Pharmaceutical Ingredient (API) (Guo et al. 2021; Kavanagh et al. 2019).

Cocrystals are multicomponent solid crystalline forms, comprising an API and a neutral coformer, stabilized through noncovalent interactions (Kumar and Nanda 2017). The intermolecular interactions and crystal packing within the crystal lattice significantly influence the physicochemical properties of the resulting solid form. In addition, incorporation of a coformer alters the molecular environment of the API, which may affect chemical stability by modifying molecular mobility through intermolecular interactions, shielding reactive functional groups, or altering their exposure at the crystal surface to environmental stress factors (Shan and Zaworotko 2008; Schultheiss and Newman 2009).

The stability of newly prepared forms is a major concern in the pharmaceutical development, as it influences subsequent manufacturing processes. Forced degradation studies are commonly designed to assess chemical stability under accelerated stress conditions, such as elevated temperature, humidity, light exposure, oxidative environment, or extreme pH (Loftsson 2014; Baertschi et al. 2016). For cannabinoid cocrystals intended for solid dosage forms, solid-state stressing is particularly relevant because it can reveal degradation products and pathways specific to the solid state, which may not be observed in solution-based stressing (Petřík et al. 2023; 2021; Maheshwari et al. 2018).

Several studies have investigated cannabinoid stability, particularly that of CBD. CBD is susceptible to degradation under light exposure (Bini et al. 2024; Seccamani et al. 2021), heat and acidic or basic environment (Fraguas-Sanchez et al. 2020; Jaidee et al. 2021; Mechoulam and Hanuš 2002; Yangsud et al. 2021). Upon irradiation, CBD shows more pronounced degradation while stored in solution (Bini et al. 2024; Seccamani et al. 2021) compared to the powder state (Kosović et al. 2021). The influence of heat and humidity has been studied for CBD solutions (Franco et al. 2022; Scheidweiler et al. 2017) and oil-based formulations (Zheng et al. 2022; Tran et al. 2023). These studies have provided valuable insights into their stability, with oil-based formulations generally less stable than solutions (Fraguas-Sanchez et al. 2020). In contrast, information regarding CBD in the powder state remains limited. The stability of CBD salts is strongly affected by their high sensitivity to heat and moisture, which requires storage at lower temperatures and in a nitrogen atmosphere (Guruprasad Reddy et al. 2023). The limited data available for CBD powder suggest that thermal stress has a greater impact on stability than by humidity (Kosović et al. 2021).

In contrast to CBD, the stability of CBN and CBG has not been extensively investigated. Available studies focus on CBN isopropanol solutions, in which photodegradation, pH-dependent oxidative stability, and thermal stability have been evaluated in comparison with CBD and THC (Tetrahydrocannabinol). Under these conditions, CBN was found to be more stable than CBD in solution (Yangsud et al. 2021). Other studies report the stability of CBN in encapsulated formulations (Villate et al. 2023), oil emulsions based on hemp seed oil, which also contain THC (Wang et al. 2024) or cannabis resins (Lindholst 2010). For CBG, existing studies mainly address long-term storage of cannabis resin and extracts, where CBG remained stable after 4 years of storage at − 20 °C (Lindholst 2010). Other studies report the stability of CBG in oral fluids (Scheidweiler et al. 2017) or photostability comparison of CBD, CBN, and CBG, where CBG and CBD remained stable after 24 h of exposure to UVA (Vacek et al. 2021). Overall, these studies provide only limited information regarding the stability of the cannabinoids, particularly in the solid state.

In our previous work, we reported several cocrystals of CBD (Koryťáková et al. 2025), CBG (Zmeškalová et al. 2025) and CBN (Koryt’áková et al. 2025), including their structural characterization and selected physicochemical properties. For CBD cocrystals, additional studies on dynamic vapour sorption and thermal behaviour have also been reported (Koryťáková et al. 2025). In addition to these known phases, the present study introduces several previously unreported CBN cocrystals, namely CBN-4,4’-bipyridine (BP), CBN-tetramethylpyrazine (TMP) (1:1) form I, CBN-TMP (1:1) form II, and CBN-TMP 2:1.

The main objective of this study is to investigate the solid-state stability of cannabinoids and their cocrystals under forced degradation conditions, with focus on the relationship between stability and solid-state structural features, such as crystal morphology and the exposure of functional groups on crystal surface. Both previously reported and newly prepared cocrystals, together with the corresponding pure cannabinoids, were subjected to a series of stress conditions, including thermal, pH-dependent, oxidative, and photolytic degradation. This approach enables a direct comparison of the degradation behaviour of the pure compounds and their cocrystals and provides insight into how cocrystal formation and crystal structure influence the stability of cannabinoids.

## Materials and methods

### Materials

Cannabidiol was purchased from PharmaHemp d.o.o (Ljubljana, Slovenia). Cannabigerol and cannabinol were purchased from Pharmabinoid B.V. (Uden, Netherlands). All of them were used as received.

The coformers 4,4′-bipyridine; L-carnitine; piperazine; L-proline; tetramethylpyrazine were purchased from Sigma-Aldrich (St. Louis, USA) and were used as received. All coformers have purity higher than 98%.

The stressing agents used for forced degradation study were Aeroperl® 300, calcium silicate, were both purchased from Evonik (Essen, Germany), Syloid® 72 FP and Syloid® 244 FP, were both purchased from Grace (Worms, Germany), Peroxydone™ XL-10 (subsequently referred to as Peroxydone) was purchased from Ashland (Wilmington, USA).

### Characterization of the stability samples

#### Powder X-ray diffraction (XRPD)

The diffraction patterns were obtained for the prepared and starting materials by a powder diffractometer X’Pert^3^ Powder (PANanalytical, Holland) equipped with Cu anode Kα (λ = 1.542 Å) with a tube voltage of 40 kV and a tube current of 30 mA. The samples were measured from 4 to 40° 2θ with 0.026° 2θ step size and 56.87 s per step.

#### Dynamic vapour sorption analysis (DVS)

Dynamic vapour sorption measurements for all phases and pure APIs were measured using SPS23-100n (ProUmid GmbH & Co. KG, Germany). The samples underwent a sorption cycle in which relative humidity was gradually increased from 0 to 90% in steps of 10%. The weighing intervals were set at 15 min. The duration at each humidity step ranged from 180 min to 10 h, depending on the time required for the sample to reach equilibrium. All measurements were conducted at 25 °C.

### Forced degradation study

#### Preparation of the samples

##### Thermal stability

Samples for thermal stability were prepared by weighing ca. 50 mg of the pure API or cocrystal in the powder state into 2 ml dark glass vials. Then, the samples were placed into the dark stability chambers set to the appropriate conditions. All powders were stored at 40 °C and 75% relative humidity (RH) for 25 days, at 60 °C and 40% or 75% RH for 13 days, and at 80 °C and 40% or 75% RH for 3 days. All the samples were prepared in duplicate for each condition. After a specific time for each condition, the samples were taken from the chambers and analysed using Ultra Performance Liquid Chromatography (UPLC) and XRPD. The samples for UPLC measurement were diluted in methanol to a final concentration of 1 mg ml^−1^. As a control sample, the non-stressed sample was used.

##### pH-dependent stability

Samples for pH-dependent stability were prepared by weighing ca. 10 mg of the powder and mixing it with pH-adjusted silica at a 1:9 ratio (API:silica, w/w). The types of silica used and their corresponding pH values are shown in Table 1. The mixtures were vortexed for 1 min at 2800 rpm and subsequently placed in dark stability chambers, where they were stressed at 60 °C and 75% RH for 13 days. All samples were prepared in duplicate. Samples were collected at 4, 8, and 13 days. After removing the samples from the chamber, each sample was diluted in methanol to a final concentration of 1 mg ml^−1^, then they were centrifuged to remove residual silica particles and analysed using UPLC. A pure sample stored at the same conditions was used as a control.

**Table 1:** Stressing agents used for pH-dependent stability with their pH values

Stressing agent	pH
Aeroperl® 300	3.9
Syloid® 72 FP	5.9
Syloid® 244 FP	6.7
Calcium silicate	9.7

##### Photostability

The photostability study was conducted in accordance with ICH Q1B guidelines (ICH Q1B Photostability testing of new active substances and medicinal products - Scientific guideline 2025). Samples were prepared by weighing ca. 40 mg of the pure API or cocrystal in powder state into a PVC sack, ensuring a sample thickness of about 1 mm. All the powders were prepared in duplicate. The samples were placed in a light-stress chamber (20 °C, 45% RH) to achieve an overall illumination exceeding 1.2 million lux/h and an integrated near-ultraviolet energy greater than 200 Wh m^−2^. After removing the samples from the stability chamber, they were diluted in methanol to a final concentration of 1 mg ml^−1^ and analysed using UPLC and XRPD. As a control sample, the sample covered with aluminium foil stored in the same stress chamber was used.

##### Oxidative stability

Oxidative stability was investigated using two different stressing agents targeting different oxidative mechanisms. Azobisisobutyronitrile (AIBN) was used as a radical initiator to induce free-radical-mediated oxidation (autooxidation), whereas Peroxydone mediates the interaction of H_2_O_2_ with the sample, which is enabling evaluation of peroxide-driven oxidation.

**Autooxidation**

Samples for the oxidative stability, autooxidation-mediated, were prepared by weighing ca. 10 mg of the powder and mixing it with mesoporous silica particles (Syloid® 72 FP) loaded with AIBN, prepared according to the publication by Petřík et al*.* (2023), in a 1:9 ratio (API:stressing agent, w/w). The mixtures were mixed for 1 min at 2800 rpm using a vortex and subsequently placed in dark stability chambers, where they were stressed at 50 °C and 75% RH for 1 day. All samples were prepared in duplicate. After removing the samples from the chamber, each sample was diluted in methanol to a final concentration 1 mg ml^−1^, then they were centrifuged to remove residual silica particles and analysed using UPLC. A control sample was prepared by mixing the API with pure silica particles (Syloid® 72 FP) and stored at the same conditions.

**Peroxide oxidation**

Samples for the oxidative stability, peroxide mediated, were prepared by weighing ca. 10 mg of the powder and mixing a complex of polyvinylpyrrolidone with H_2_O_2_ (Peroxydone), which was prepared according to publication of Modhave et al*.* (2019), in a 1:9 ratio (API:stressing agent, w/w). The mixtures were mixed for 1 min at 2800 rpm using a vortex and subsequently placed in dark stability chambers, where they were stressed at 40 °C and 50% RH for 5 days. All samples were prepared in duplicate. After removing the samples from the chamber, each sample was diluted in methanol to a final concentration of 1 mg ml^−1^, followed by centrifugation to remove residual silica particles. A pure sample stored under the same conditions was used as a control.

#### UPLC purity analysis

The UPLC analysis was performed using 1290 Infinity II LC System (Agilent Technologies Inc., USA) with a high-pressure pump, an autosampler, a thermostat and a diode array detector. An Acquity UPLC CSH Phenyl-Hexyl column (Waters Corporation, USA) with dimensions of 100 mm length and 2.1 mm internal diameter with particle size of 1.7 μm was used for the purity analysis. The autosampler temperature was set to 5 °C, and the column heater was set to 20 °C during analysis. For the separation, ultrapure water and acetonitrile were used as components A and B, respectively, of the mobile phase. The gradient elution was set as follows: *t* (min)/*%* B: 0/50; 12/75; 14/100; 20/100; 20.5/50; 24/50. The flow rate of the mobile phase was 0.3 ml min^−1^. The injection volume was set to 2 μl per sample. The detection was carried out at 238 nm for CBD and CBG samples and 284 nm for CBN samples using the UV/Vis detector. The chromatographic data were evaluated using OpenLab CDS software. The purity was determined using the method of internal normalisation of chromatographic peak areas.

### Calculation procedures

#### Morphology prediction

Crystal morphologies were predicted using the VisualHabit model with the Dreiding II force field and a limiting radius of 30 Å implemented in the CSD-Particle module (Moldovan and Maloney 2024; Prandini et al. 2024) of Mercury (version 2025.1.1, build 448738) (Macrae et al. 2020).

#### Calculation of conformational strain energy

Conformational strain energies were determined using DFT in Gaussian 16, employing the B3LYP functional with D3BJ dispersion correction and the 6-311G(d,p) basis set. For each polymorph, a single CBN molecule was extracted from the crystal structure in Mercury. A constrained geometry optimization was then performed, in which the torsion angles were fixed to preserve the crystal conformation, while bond lengths and bond angles were allowed to relax. Subsequently, the same molecule was fully optimized in the gas phase without any constraints at the same level of theory (Bhardwaj et al. 2019). The conformational strain energy was calculated according to Eq. (1):

1 $$\begin{array}{c}{\Delta E}_{\mathrm{s}\mathrm{t}\mathrm{r}\mathrm{a}\mathrm{i}\mathrm{n}}= {E}_{\mathrm{c}\mathrm{r}\mathrm{y}\mathrm{s}\mathrm{t}\mathrm{a}\mathrm{l}}-{E}_{\mathrm{g}\mathrm{a}\mathrm{s} \mathrm{p}\mathrm{h}\mathrm{a}\mathrm{s}\mathrm{e}} \end{array}$$

where ${E}_{\mathrm{c}\mathrm{r}\mathrm{y}\mathrm{s}\mathrm{t}\mathrm{a}\mathrm{l}}$ corresponds to the energy of the constrained molecule of the crystal, and ${E}_{\mathrm{g}\mathrm{a}\mathrm{s} \mathrm{p}\mathrm{h}\mathrm{a}\mathrm{s}\mathrm{e}}$ is the energy of the fully relaxed gas-phase molecule. Energies obtained in Hartree were converted to kJ mol^−1^ using the conversion factor 1 Hartree = 2625.5 kJ mol^−1^.

#### Particle energy calculations

The particle energy was calculated using the following Eq. (2) (Sacchi et al. 2024):

2 $$\begin{aligned}{E}_{\mathrm{p}\mathrm{a}\mathrm{r}\mathrm{t}\mathrm{i}\mathrm{c}\mathrm{l}\mathrm{e}}&= {E}_{\mathrm{l}\mathrm{a}\mathrm{t}\mathrm{t}\mathrm{i}\mathrm{c}\mathrm{e}(\mathrm{i}\mathrm{n}\mathrm{t}\mathrm{e}\mathrm{r})}+{\Delta E}_{\mathrm{c}\mathrm{o}\mathrm{n}\mathrm{f}\mathrm{o}\mathrm{r}\mathrm{m}\mathrm{a}\mathrm{t}\mathrm{i}\mathrm{o}\mathrm{n}\mathrm{a}\mathrm{l}(\mathrm{i}\mathrm{n}\mathrm{t}\mathrm{r}\mathrm{a})}\\& \quad-0.5 \sum {x}_{\left(\mathrm{h}\mathrm{k}\mathrm{l}\right)} {E}_{\left(\mathrm{h}\mathrm{k}\mathrm{l}\right)}\end{aligned}$$

The first term represents the lattice energy, describing the strength of intermolecular interactions that stabilize the bulk crystal. The conformational energy term represents the energetic cost for a molecule to adopt the specific conformation, which is required within the crystal lattice, relative to its lowest energy reference state. The final term accounts for surface energy, describing how the termination of crystal faces impacts particle stability. This contribution involves the fractional surface area of each exposed crystal face and the corresponding attachment energy associated with.

## Results and discussion

The forced degradation study comprises all available cannabinoid cocrystals: CBD-BP, CBD-L-carnitine (CR), CBD-PI, CBD-L-proline (PR), CBD-TMP; CBN-BP, CBN-PI form III, CBN-TMP (1:1) form I and II, CBN-TMP 2:1; CBG-PI and CBG-TMP form I, together with the corresponding pure APIs (CBD, CBN, and CBG). In addition, several newly obtained CBN cocrystals with BP and TMP as coformers are described in Section SI 1. These new phases were structurally characterized and their physicochemical properties, such as thermal and dissolution behaviour, were determined in Section SI 2.

### Crystal morphology and surface features

Crystal morphology describes the shape and dominant facets of crystals, in addition, it provides insight into the functional groups exposed on the crystal surfaces. Differences between crystal forms reflect variations in crystal packing and intermolecular interactions, which influence physicochemical properties such as dissolution behaviour (Zmeškalová et al. 2025), stability of polymorphs (Koryt’áková et al. 2025), mechanical strength (Bryant et al. 2019) and water sorption (Thakuria et al. 2019). Furthermore, the nature and accessibility of functional groups exposed on crystal surfaces may influence their interaction with external agents such as moisture, oxygen and reactive species (Dhondale et al. 2023). However, surface exposure alone does not determine reactivity, as intermolecular interactions and crystal packing can also affect the accessibility of chemically labile sites.

**Fig. 1:**
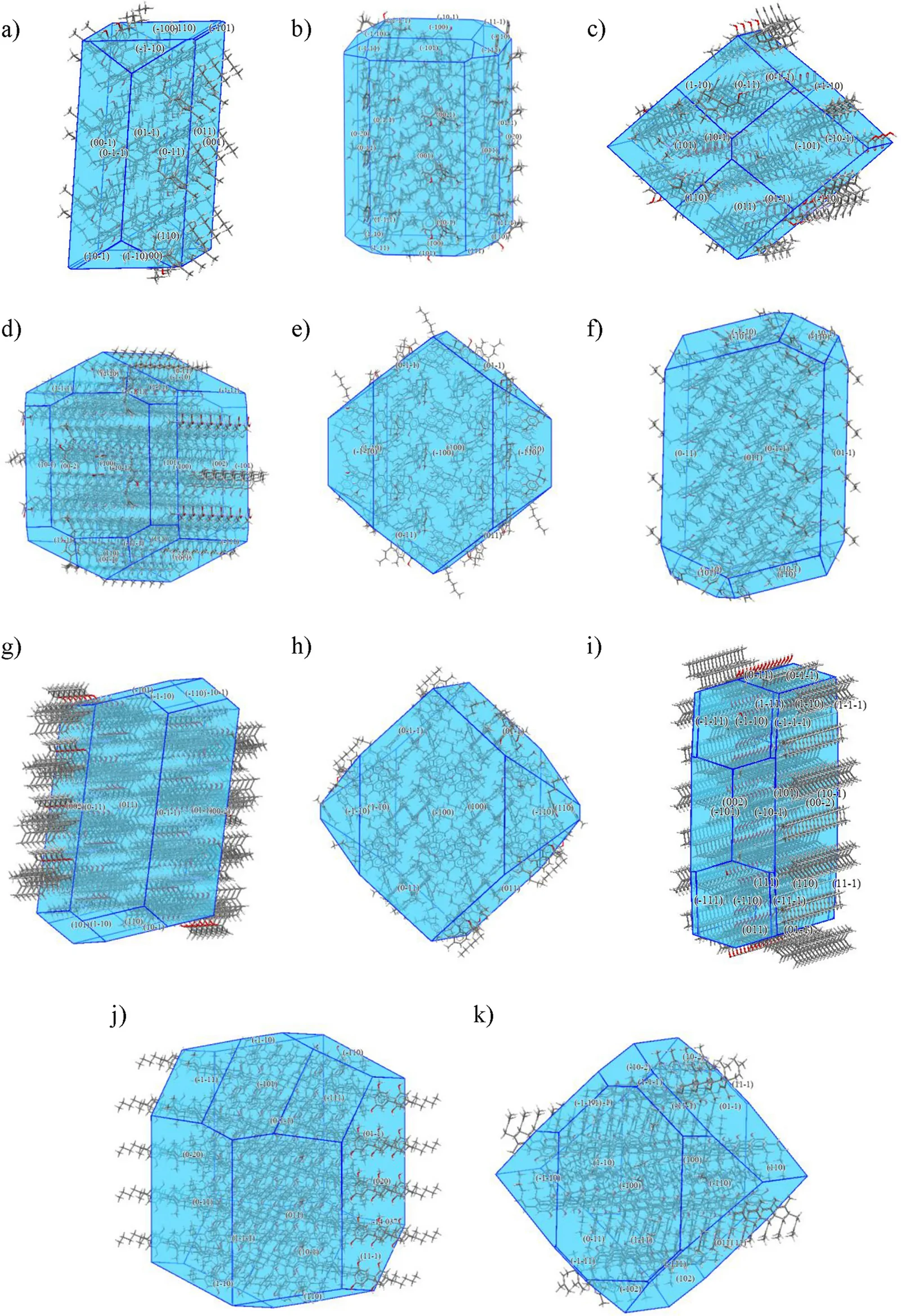
Comparison of predicted morphologies of **a**) CBD, **b**) CBD-BP, **c**) CBD-CR, **d**) CBD-PR, **e**) CBN, **f**) CBN-BP, **g**) CBN-PI form III, **h**) CBN-TMP 2:1, **i**) CBG **j**) CBG-PI, and **k**) CBG-TMP form I

In our previous study we used the predicted morphologies to support and further explain the results of dynamic vapour sorption and the thermal stability of CBD cocrystals (Koryťáková et al. 2025). In the present work, this approach is extended to CBN and CBG cocrystals, and the morphology predictions of all investigated cocrystals and pure cannabinoids are summarized in Fig. 1 for direct comparison. The predicted morphologies for the already published CBG cocrystals (Zmeškalová et al. 2025) and CBN cocrystals are discussed in more detail in SI 3 and SI 2.1.2.

The crystal facets differ among the studied solid forms, reflecting variations in crystal packing, intermolecular interactions, and the spatial arrangement of functional groups. These variations influence the surface exposure of functional groups, thereby modifying the physicochemical properties of the systems. Functional groups that are exposed and weakly bond within the crystal lattice are more accessible to reactive species and can participate in degradation processes such as oxidation or hydrolysis. In contrast, groups involved in strong intermolecular interactions or located within densely packed regions of the crystal are less accessible and therefore less prone to degradation. Such structural variations are considered in the following section to interpret and rationalize the stability behaviour under different stress conditions and to establish structure-stability relationships among the investigated solid forms.

### Dynamic vapour sorption

**Fig. 2:**
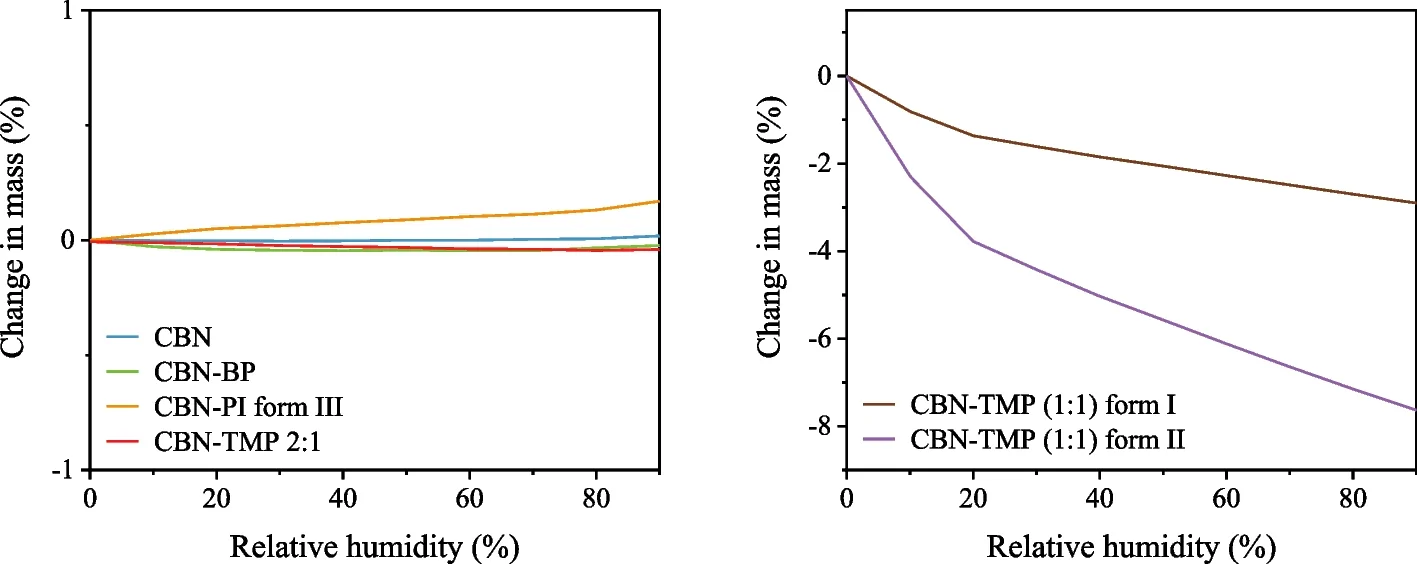
The DVS curves of CBN and its cocrystals

**Fig. 3:**
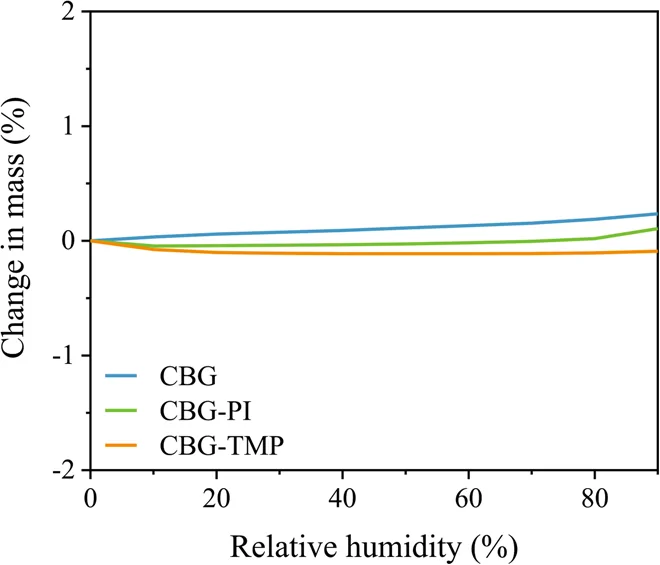
The DVS curves of CBG and its cocrystals

The DVS analysis of the CBD cocrystals has been discussed in our previous work (Koryťáková et al. 2025). It was shown that the stability of the CBD cocrystals under moisture conditions is influenced by their crystal morphology and the functional groups exposed on their surfaces (Koryťáková et al. 2025; Thakuria et al. 2019). Furthermore, we extend the DVS analysis to the CBN (Fig. 2) and CBG (Fig. 3) cocrystals, which have not yet been evaluated.

Pure CBN exhibits low moisture uptake even at higher relative humidity, which is consistent with its hydrophobic nature (Khouchlaa et al. 2024). Similar hydrophobic behaviour was observed for CBN-BP, CBN-PI form III, and CBN-TMP 2:1. These DVS results correlate well with the predicted morphologies (Fig. 1e, f, g, h), which show that the hydrophobic groups are mostly exposed on the crystal faces.

In contrast, both polymorphic forms of CBN-TMP (1:1) exhibited increasing weight loss with increasing humidity. A comparable behaviour was previously observed for CBD-TMP cocrystal (Koryťáková et al. 2025) and it was connected to the tendency of tetramethylpyrazine to sublimate (Xie et al. 2024). The weight loss for CBD-TMP was approximately 0.7%. However, in the case of CBN-TMP (1:1) in forms I and II, the weight loss was significantly higher, 2.9% and 7.6%, respectively, indicating instability of these cocrystals.

To investigate further the decrease in mass of CBN-TMP cocrystals, we performed XRPD analysis after the DVS experiment (Figure SI 19). In the case of form I, the XRPD pattern revealed a partial conversion to the CBN-TMP 2:1 cocrystal; a dominant peak of this cocrystal at 8.02° 2*θ* was observed, while characteristic peaks of the original form I (e.g., 7.04, 8.82° 2*θ*) remained present. In contrast, form II underwent a complete conversion to the CBN-TMP 2:1 cocrystal. These transformations are probably driven by the instability of the 1:1 cocrystal, which results in the formation of a more stable 2:1 cocrystal.

The observed transformation behaviour can be supported by calculated interaction energies, which show that CBN-TMP (1:1) form II exhibits a lower interaction energy (− 48.1 kJ mol^−1^, Figure SI 7b) than form I (− 53.4 kJ mol^−1^, Figure SI 6b), while the CBN-TMP 2:1 cocrystal exhibits the highest interaction energy (− 54.0 kJ mol^−1^, Figure SI 8b). In addition, the particle energy was calculated for the polymorphs to explain the partial and full conversion to the cocrystal in a 2:1 ratio. The calculations (Table SI 3) revealed that form II has a lower particle energy (− 106.4 kJ mol^−1^) than form I (− 111.1 kJ mol^−1^), which indicates a higher stability of form I. Therefore, form II may more easily undergo full conversion to the 2:1 cocrystal, whereas form I is only partially converted. Overall, the instability of the cocrystals in a 1:1 ratio is probably driven by the tendency of TMP to sublimate even under ambient conditions (Xie et al. 2024), together with the crystal arrangement and intermolecular interactions.

In addition, we conducted time-dependent XRPD measurements (Figure SI 20) to investigate the transformation of form I and II under ambient conditions. These results revealed that the CBN-TMP 2:1 phase becomes dominant within 48 h, with the full conversion occurring within 72 h. Due to their instability, forms I and II of CBN-TMP cocrystal in a 1:1 ratio were not included in subsequent experiments.

The DVS analysis of CBG and its cocrystals showed negligible moisture uptake throughout the experiment. Pure CBG showed only a slight, gradual increase in water sorption at high RH, consistent with its hydrophobic character (Peters et al. 2023). This behaviour correlates well with the predicted morphology (Fig. 1i), which showed that on the dominant surfaces are exposed hydrophobic groups, with only minimal exposure of hydroxyl groups. A comparable behaviour was observed for the CBG-PI cocrystal, where the morphology (Fig. 1j) shows mostly hydrophobic aliphatic tails of CBG. The minor increase in vapour sorption at elevated RH levels can be attributed to the higher hygroscopic tendency of piperazine (Wang et al. 2019), a behaviour previously observed for the CBD-PI cocrystal (Koryťáková et al. 2025) or the exposure of hydroxyl groups on the largest surfaces. In addition, an initial decrease in sample mass was observed at the beginning of the sorption cycle for both CBG-PI and CBG-TMP form I. This mass loss is likely associated with partial sublimation of PI (Wang et al. 2019) and TMP (Xie et al. 2024), which undergo sublimation under ambient conditions, and a similar behaviour was observed in case of CBD (Koryťáková et al. 2025) and CBN cocrystals. Furthermore, the observed hydrophobic behaviour of CBG-TMP form I is consistent with the predicted morphology (Fig. 1k), where the dominant faces are composed of hydrophobic groups (aliphatic tails of CBG).

Overall, the DVS results show that differences in the crystal packing and surface composition influence the accessibility of water molecules to the crystal surfaces. Consequently, these structural features may contribute to the different stability behaviour under moisture-containing stress conditions.

### Forced degradation study

The forced degradation study was performed on the cannabinoid (CBD, CBN and CBG) cocrystals under various stress conditions, including thermal degradation, pH-dependent, oxidative and photostability stressing. For each sample, the API amount was analysed using UPLC. Selected representative UPLC chromatograms obtained for non-stressed and stressed samples of pure cannabinoids are provided in Section SI 5.1. Additionally, visual changes, such as colour and state of the powder were checked and the crystalline state of samples of the thermal and photostability studies was evaluated using XRPD after removal from the stability chambers. In the pH-dependent and oxidative stability studies, the XRPD measurement did not provide any information because the XRPD patterns were dominated by the stressing agent. All the samples before the accelerated stress study were white powders, except CBD-PI, which was originally brownish.

#### Thermal stability

The thermal degradation study was conducted first, as temperature is a critical factor for cannabinoids, which are generally low-melting compounds. The thermal stability of CBD and its cocrystals has been reported previously (Koryťáková et al. 2025). Therefore, we focused on the thermal stability of CBG and CBN, and their cocrystals.

##### CBN and its cocrystals

**Fig. 4:**
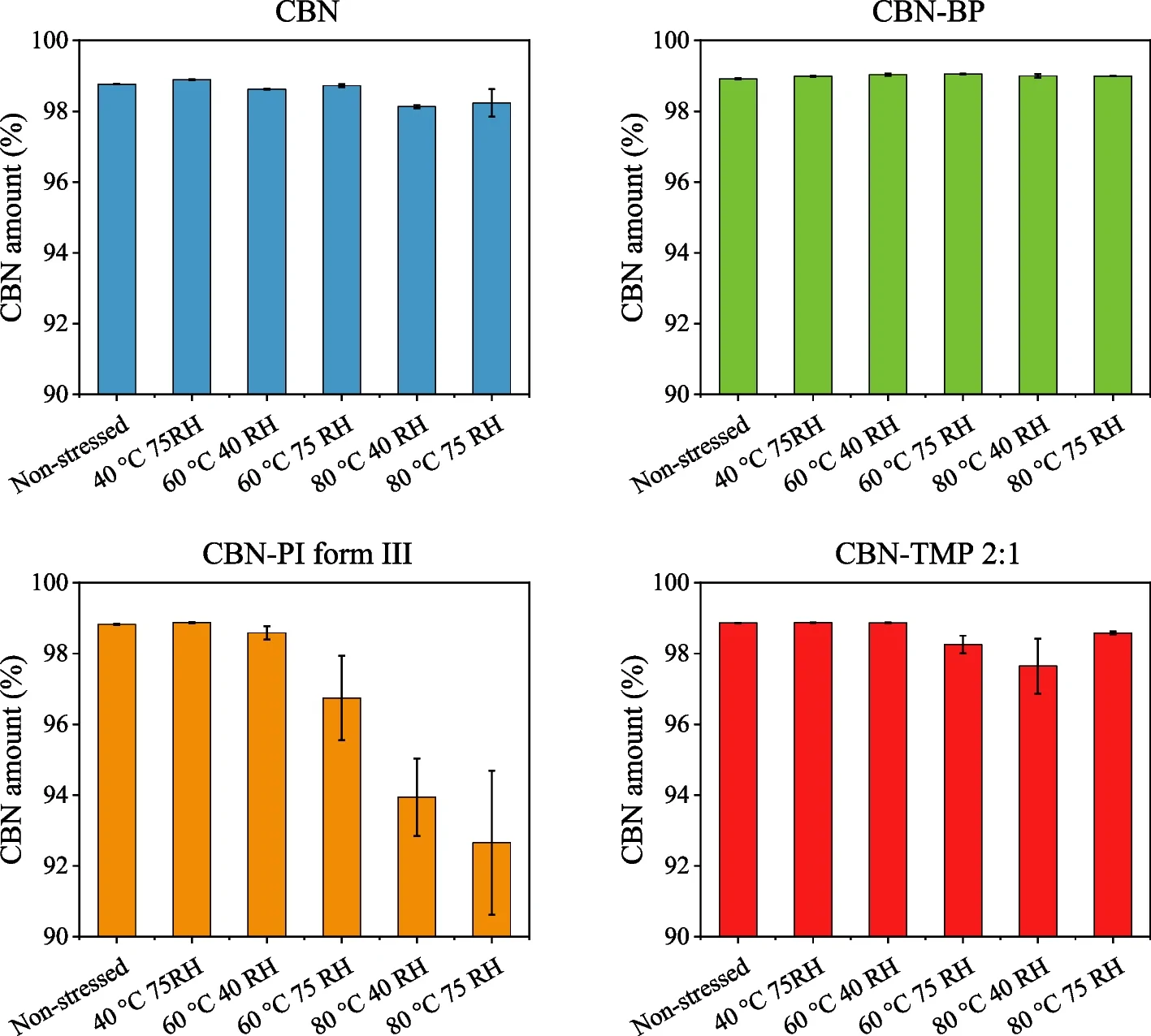
The CBN amount in non-stressed cocrystals and pure CBN and after exposure to thermal stress

The thermal stability results for CBN cocrystals and pure CBN are shown in Fig. 4. Pure CBN remained stable when stored at 40 °C/75% RH and 60 °C/75% RH, which was confirmed by both UPLC and XRPD analysis (Figure SI 39a). The sample stored at 60 °C/40% RH also did not show a significant difference in CBD amount; however, it changed to a jelly-like state with a brownish colour. In contrast, the samples stored at 80 °C showed a considerable difference in the CBN amount. Furthermore, the samples were changed into a jelly-like state with a brownish colour, probably because the storage conditions were close to or even exceeded the melting point of CBN.

The CBN-BP cocrystal showed no decrease in CBN amount and it remained stable throughout the entire time of the experiment under all tested conditions. Moreover, the crystalline state did not undergo any transformation, as confirmed by XRPD (Figure SI 39b), and all samples were powders after removal from the stability chambers.

The CBN amount in the CBN-PI form III sample remained stable when stored at 40 °C/75% RH; moreover, the crystalline state was preserved, as indicated by XRPD analysis (Figure SI 39c). In contrast, storage under the remaining conditions led to a significant decrease in the CBN amount. For the samples stored at 60 °C, the decrease in CBN amount was less pronounced within the experimental error. XRPD analysis showed that the crystalline state remained unchanged, although the sample colour changed to brownish, which can be attributed to surface oxidation of the cocrystal. The samples stored at 80 °C became jelly-like, probably because the storage temperature was close to the cocrystal's melting point.

CBN-TMP 2:1 cocrystal showed no decrease in CBN amount when stored at 40 °C/75% RH and 60 °C/40% RH, and the XRPD analysis (Figure SI 39 d) confirmed the crystalline state remained unchanged. In contrast, storage at 60 °C/75% RH resulted in partial melting of the material, as evidenced by changes in the XRPD diffractogram. The XRPD pattern showed new peaks, e.g., at 5.88, 9.91, and 11.37° 2*θ*, corresponding to the pure CBN. However, the peaks corresponding to the cocrystal are still present (e.g., 8.06, 10.54, 13.84° 2*θ*). The peaks corresponding to pure TMP are absent, as TMP tends to sublimate even at ambient conditions (Xie et al. 2024). This observation suggests partial disintegration of the cocrystal into the initial compounds. Both samples stored at 80 °C melted, consistent with the storage temperature exceeding the cocrystal's melting point.

In conclusion, the CBN-BP exhibited with improved stability than the rest of the cocrystals. It remained in a powder state throughout the entire storage period. The higher stability may be caused by the fact that the morphology prediction (Fig. 1f) shows mostly hydrophobic parts on the surface. However, in case of CBN cocrystals, we did not observe any correlation between interaction energy and tendency to degrade as for CBD cocrystals (Koryťáková et al. 2025). For example, the CBN-BP exhibited the lowest interaction energy (− 43.3 kJ mol^−1^), yet it was the most stable cocrystal. In contrast, CBN-PI form III exhibited the highest interaction energy (− 64.0 kJ mol^−1^) but also the highest instability. For CBN-PI form III and CBN-TMP 2:1, the higher instability may be related to the nature of the coformers, which are more exposed on the crystal surfaces (Fig. 1g, h), which may facilitate interaction with atmospheric moisture and decrease the cocrystal stability.

##### CBG and its cocrystals

**Fig. 5:**
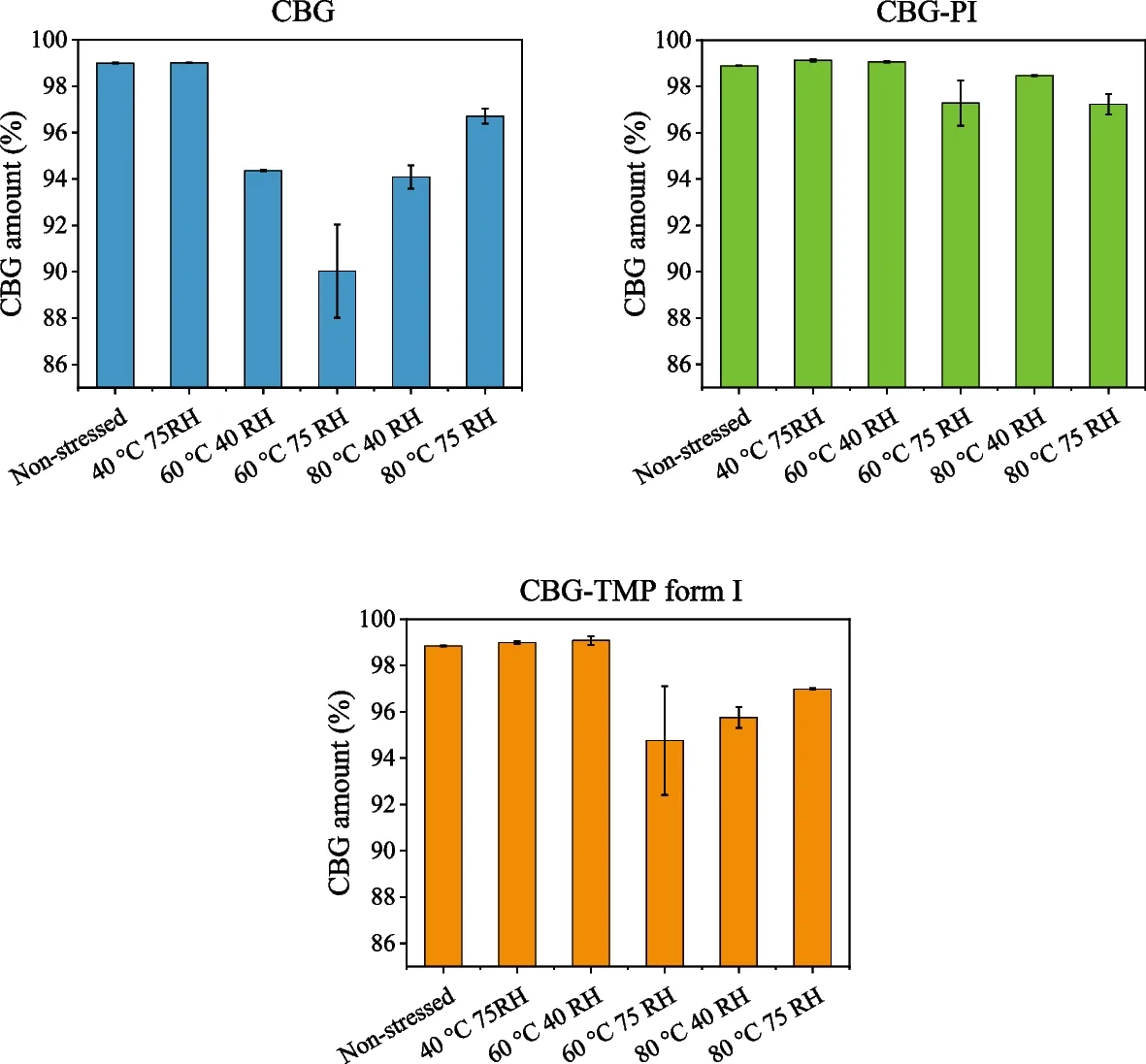
The CBG amount in non-stressed cocrystals and pure CBG and after exposure to thermal stress

The thermal stability results for pure CBG and its cocrystals are shown in Fig. 5. Pure CBG remained stable only while stored at 40 °C/75% RH, which was confirmed by both UPLC and XRPD analyses (Figure SI 40a). Under other conditions, we observed pronounced degradation. In addition, the samples turned jelly-like and brownish. These changes are attributed to the storage temperature exceeding the melting point of pure CBG. After removal from stability chambers, the samples were stored at 60 °C/75% and 80 °C/75 gradually dried, and subsequent XRPD analysis revealed changes in the diffraction pattern compared to the non-stressed CBG. We observed new peaks at 11.27, 13.02, and 16.84° 2θ, while characteristic peaks at 12.03, 17.85, and 18.33° 2*θ* disappeared. These changes in the XPRD pattern are consistent with the degradation detected by UPLC analysis.

No degradation of CBG-PI was observed upon storing at 40 °C and 60 °C/75 RH, and the cocrystal remained stable according to XRPD analysis (Figure SI 40b). Under the remaining conditions, a slight decrease in CBG amount was observed; however, considering the error bars, the decrease was minor. A visible colour change to beige was noted in these samples. Despite the change in appearance, XRPD analysis did not reveal any changes in the solid-state structure. This observation suggests that the colour change is likely associated with surface changes, such as oxidation, which are not detectable by XRPD, but can be identified by UPLC analysis.

CBG-TMP form I stored at 40 °C/75% RH and 60 °C/40% RH did not result in a decrease in the CBG amount. However, XRPD analysis (Figure SI 40c) revealed changes in the solid state of the cocrystal. Under both conditions, the cocrystal began to partially dissociate into the initial compounds, indicated by two characteristic peaks of pure CBG at 4.29 and 4.67° 2*θ*, while the characteristic peaks of the CBG-TMP form I cocrystal are still preserved, e.g., 9.66, 12.14 and 17.20° 2*θ*. In contrast, storing CBG-TMP form I at 60 °C/75% RH and 80 °C led to a significant decrease in CBG amount. After removal from the stability chambers, all the samples were melted and exhibited a brownish colour. These changes can be attributed to the fact that the storage temperature is close or exceeded the melting point of the cocrystal. The sample stored at 80 °C/75 gradually dried, and subsequent XRPD analysis revealed almost complete disintegration of the cocrystal, with only weak reflections at 9.66 and 17.20° 2*θ* remaining and dominant reflections corresponding to pure CBG at 4.67, 15.28 and 17.85° 2*θ* and pure TMP at 16.73 and 21.37° 2*θ* were observed.

In conclusion, the CBG-PI cocrystal exhibited enhanced stability compared to both pure CBG and the CBG-TMP form I cocrystal, remaining stable throughout the entire storage period. When comparing the calculated interaction energies of the cocrystals and pure CBG with their stability behaviour, we did not observe any correlation similar to that of CBD and its cocrystals (Koryťáková et al. 2025). Although pure CBG exhibited the highest interaction energy (− 69.2 kJ mol^−1^), it showed the lowest stability. In contrast, CBG-PI showed improved stability despite exhibiting intermediate interaction energy (− 54.7 kJ mol^−1^). The enhanced thermal stability of CBG-PI is likely related to its melting point, which is more than twice that of pure CBG, as well as the crystal surface (Fig. 1j), which consist of hydrophobic groups of CBG, while PI molecules are not exposed, unlike in the CBD and CBN cocrystal, where coformer exposure is likely reducing their stability. The higher instability of pure CBG and CBG-TMP form I is likely associated with their relatively lower melting points.

Overall, the results suggest that the thermal stability is governed by a combination of crystal packing, melting behaviour and moisture uptake rather than by interaction energy alone. Therefore, interaction energy does not appear to be the dominant factor in determining their thermal stability.

#### pH-dependent stability

To understand the pH-dependent stability of the cocrystals, we should also consider the pH-dependent behaviour of the individual components based on their functional groups.

The summarized ionization behaviour of the components is presented in Table 2. All cannabinoids remain neutral over the entire investigated pH range, as their reported pK_a_ values are 9.13 (CBD), 9.16 (CBG) and 9.4 (CBN) (Dawidowicz et al. 2021). 4,4’-bipyridine exhibits two pK_a_ values at 3.17 and 4.82, which indicates stepwise protonation of the nitrogen atoms (Krumholz 1951). Consequently, BP is expected to be partially protonated at pH 3.9 and to remain neutral at higher pH values. For L-carnitine, only limited information regarding its pK_a_ values is available; however, it has been reported,

**Table 2:** Protonation state of compounds used in CBD and CBN cocrystals at different pH values

Compound	pH 3.9	pH 5.9	pH 6.7	pH 9.7
CBD	0	0	0	⁓ 0
CBG	0	0	0	⁓ 0
CBN	0	0	0	⁓ 0
BP	+ 1	0	0	0
CR	0 (zwitterion)	0 (zwitterion)	0 (zwitterion)	⁓ 0 (zwitterion)
PI	+ 2	+ 1	+ 1	0
PR	0 (zwitterion)	0 (zwitterion)	0 (zwitterion)	0 (zwitterion)
TMP	0	0	0	0

that at pH 7.4 it exists in a zwitterionic form (Harmeyer 2002). L-proline exhibits a pK_a_ value of 1.99 for carboxylic group deprotonation and 10.6 for amine group deprotonation (Smith et al. 1942). Based on the similarities in the functional groups and amino acid nature, we can assume, that L-carnitine exhibits zwitterionic behaviour similar to that of L-proline across the investigated pH range.

Piperazine, which contains two nitrogen atoms, exhibits two pK_a_ values at 5.54 and 9.68 (Pagano et al. 1961). Therefore, we expect that piperazine is doubly protonated at pH 3.9, mono-protonated at pH 5.9 and 6.7, and neutral at the highest pH. Finally, tetramethylpyrazine exhibits two pK_a_ values, with the first at − 2.7 and the second at 3.55 (Chia and Trimble 1961). Based on these values, tetramethylpyrazine is expected to remain neutral over the studied pH range.

##### CBD and its cocrystals

**Fig. 6:**
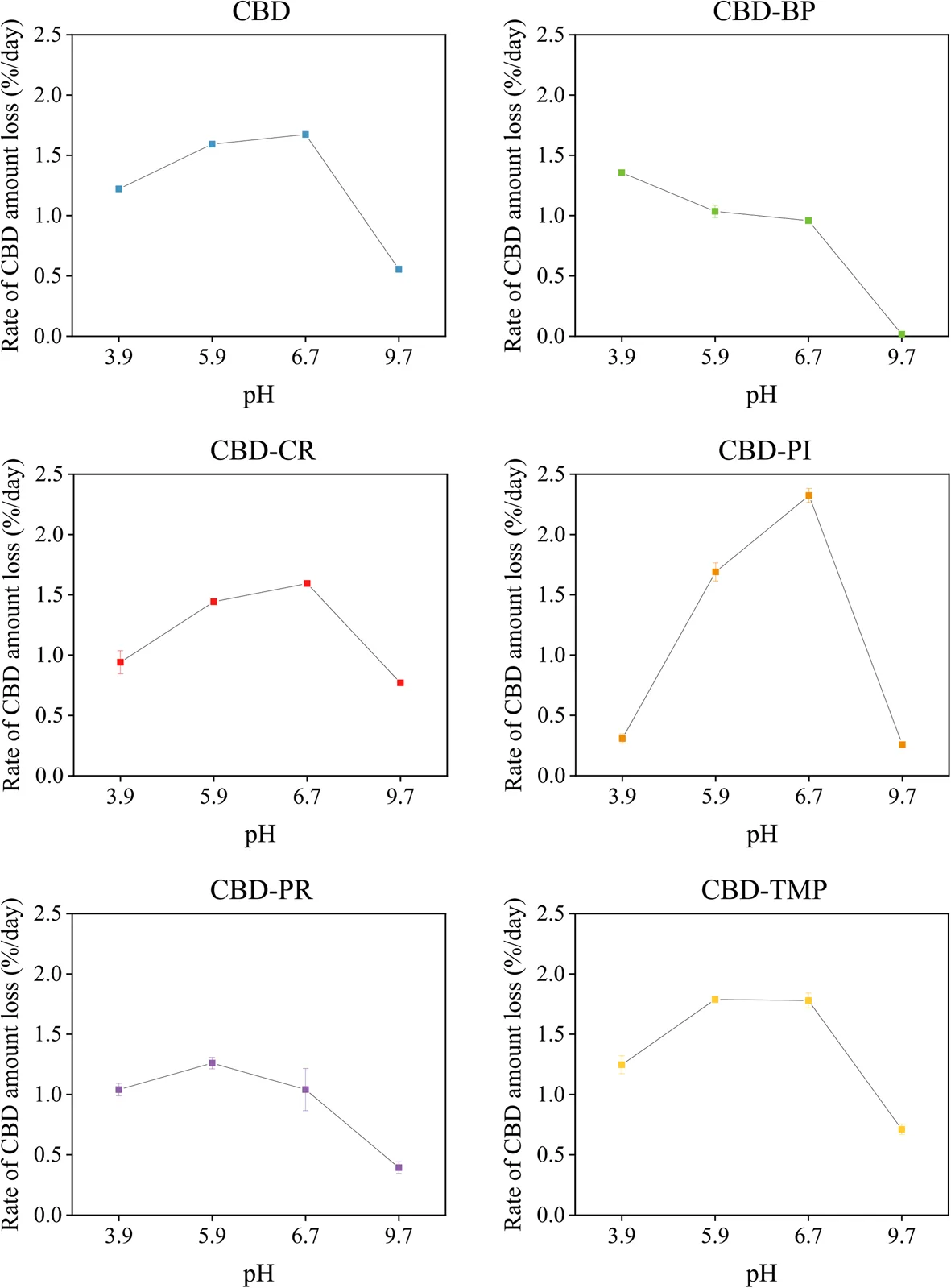
Hydrolytic curves of the pure CBD and its cocrystals

Pure CBD and all CBD cocrystals, except CBD-BP, exhibited similar trends in the hydrolytic curves (Fig. 6). The lowest and highest pH conditions resulted in lower CBD losses, whereas intermediate pH values led to higher CBD losses.

The CBD-CR and CBD-TMP showed the hydrolytic curves to be very similar to that of pure CBD, which suggests that the coformers have a minimal influence on the pH stability.

of the cocrystals. This behaviour may be related to the fact, that both coformers remain uncharged across the investigated pH range, which results in stability of the cocrystals comparable to that of pure CBD.

In contrast, the CBD-BP exhibited a different pH-dependent behaviour compared to the other cocrystals, where a decreasing rate of CBD loss with increasing pH was observed. The higher rate of CBD loss at pH 3.9 may be due to protonation of the coformer, which can influence intermolecular interactions within the cocrystal. At pH 9.7, the cocrystal showed a nearly zero rate of CBD loss, which indicates a significant stabilizing and protecting effect of this cocrystal on CBD.

The CBD-PI cocrystal exhibited similar pH-dependent behaviour as pure CBD, where low rates of CBD loss were observed at the lowest and highest pH values. In contrast, the highest rate of loss among all cocrystals and pure CBD was detected at pH 6.7. The possible reason for this behaviour may be related to the protonation state of the coformer, where the fully protonated or fully unprotonated state leads to increased cocrystal stability.

Finally, CBD-PR cocrystal exhibited enhanced stability compared to pure CBD across all investigated pH values. This suggests that L-proline shows a stabilizing effect on the CBD. However, this protective effect is less pronounced compared to that of 4,4’-bipyridine.

##### CBN and its cocrystals

**Fig. 7:**
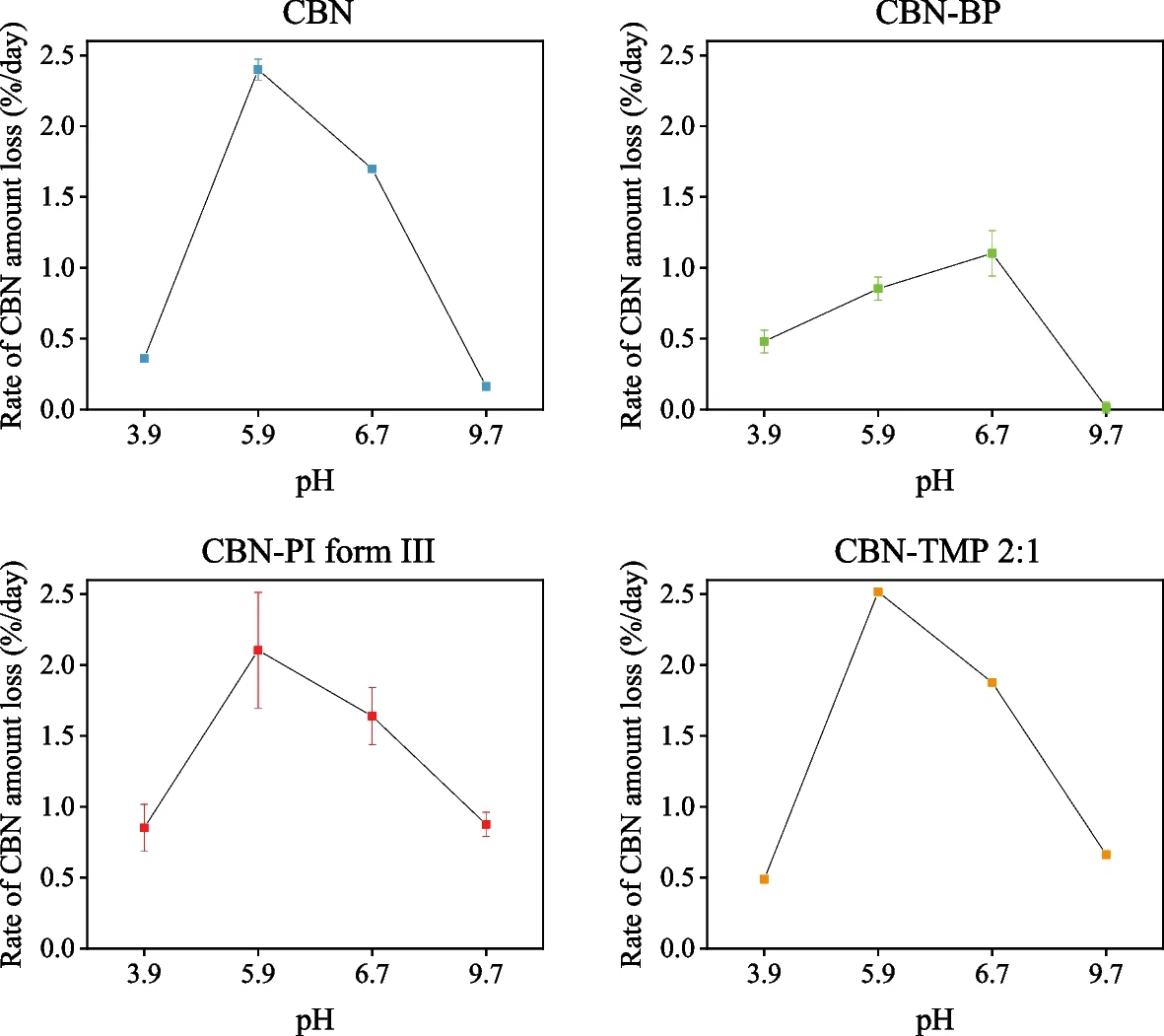
Hydrolytic curves of the pure CBN and its cocrystals

Pure CBN and its cocrystals, except CBN-BP, exhibited similar trends in hydrolytic curves (Fig. 7), when the error bars are considered. The lowest and highest pH values led to a lower rate of CBN loss, while the intermediate pH values resulted in higher CBN loss, similar to the trend of CBD and its cocrystals.

Pure CBN showed the highest rate of degradation at pH 5.9, which was approximately 1.5 times higher than that observed for pure CBD. In contrast, at the lowest and highest pH values, pure CBN exhibited lower rates of CBN loss compared to pure CBD.

The CBN-BP cocrystal exhibited a different behaviour across the investigated pH values, with the rate of CBN loss increasing with increasing pH values to pH 6.7. However, at pH 9.7, as with CBD-BP, the pH did not influence cocrystal stability. Furthermore, at pH 5.9 and 6.7, the cocrystal showed greater stability than pure CBN.

The CBN-PI form III showed, on average, a higher rate of CBN loss than CBN, suggesting that the coformer reduces cocrystal stability, likely due to its partial charge. Similar behaviour was observed for CBD-PI, where the fully protonated and unprotonated states are more favourable, whereas the mono protonated states are associated with increased instability of the cocrystal.

In contrast, CBN-TMP 2:1 exhibited a similar rate of CBN loss compared to pure CBN. This indicates that the coformer does not significantly affect the stability of the cocrystal, either positively or negatively.

##### CBG and its cocrystals

**Fig. 8:**
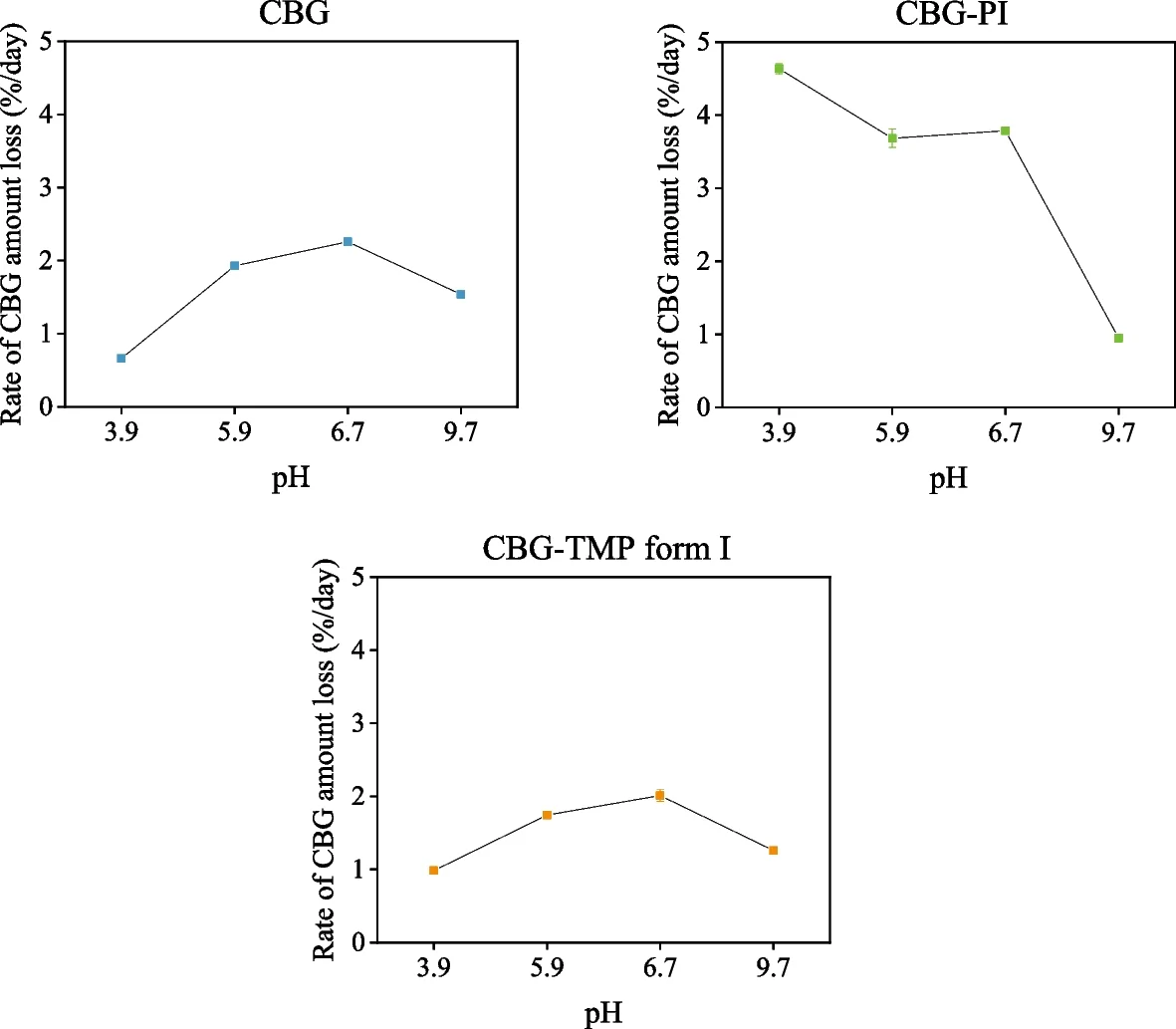
Hydrolytic curves of the pure CBG and its cocrystals

Pure CBG and CBG-TMP form I exhibited similar trends in the hydrolytic profiles (Fig. 8). As observed for CBD and CBN and their cocrystals, the lowest and highest pH values generally resulted in lower rates of CBG loss, whereas the intermediate pH values led in increased CBG loss.

Pure CBG exhibited the highest degradation rate at pH 6.7, exceeding those observed for CBD and CBN. In addition, at pH 9.7, it exhibited the highest rate of degradation among the investigated cannabinoids' solid forms.

In contrast, CBG-PI exhibited a different pH-dependent behaviour, with the rate of CBG loss decreasing as the pH increased. Overall, it showed the highest degradation rates among the investigated cannabinoids, except at pH 9.7. This behaviour may be related to its protonated state, similar to CBD-PI and CBM-PI form III. Despite its generally higher degradation rates, CBG-PI showed a lower rate of CBG loss at pH 9.7 compared to pure CBG, which indicates a protective effect on the CBG.

The CBG-TMP form I showed similar behaviour as pure CBG across the investigated pH range, with slightly lower degradation rates. However, no significant stabilizing effect of the coformer on the CBG was observed.

##### Summary of pH-dependent studies

Overall, CBD-BP, CBN-BP, and CBD-PR showed improved stability across different pH environments compared to the pure APIs, suggesting a protective effect of the cocrystal on the APIs. CBG-PI showed improved stability only at pH 9.7 compared to pure CBG. However, the CBD-PI, CBN-PI form III, and CBG-PI cocrystals exhibited higher rates of API loss, which may be related to the partial charge of the coformer, which may accelerate the cocrystal instability.

In general, the hydrolytic curves of the cocrystals were, in most cases, comparable to those.

of the pure APIs. Furthermore, we observed a consistent trend, where the lowest and highest pH values resulted in lower rates of API loss, while intermediate pH values were connected with the highest rates of API loss. Moreover, the rate of CBD and CBG loss for pure CBD and CBG was flatter, with negligible differences across almost all investigated pH ranges, in contrast to pure CBN, where the lowest rates of loss were approximately six times lower than the highest ones. For CBD and CBG, this difference was approximately 3.5-fold. The highest pH value (pH 9.7) was associated with the greatest stability across all investigated cocrystals and pure API. Furthermore, we observed similar stability trends for cocrystals containing the same coformer, indicating that the choice of coformer, particularly the presence of ionizable functional groups, can influence cocrystal stability and, consequently, API degradation under different pH conditions.

#### Photostability

**Fig. 9:**
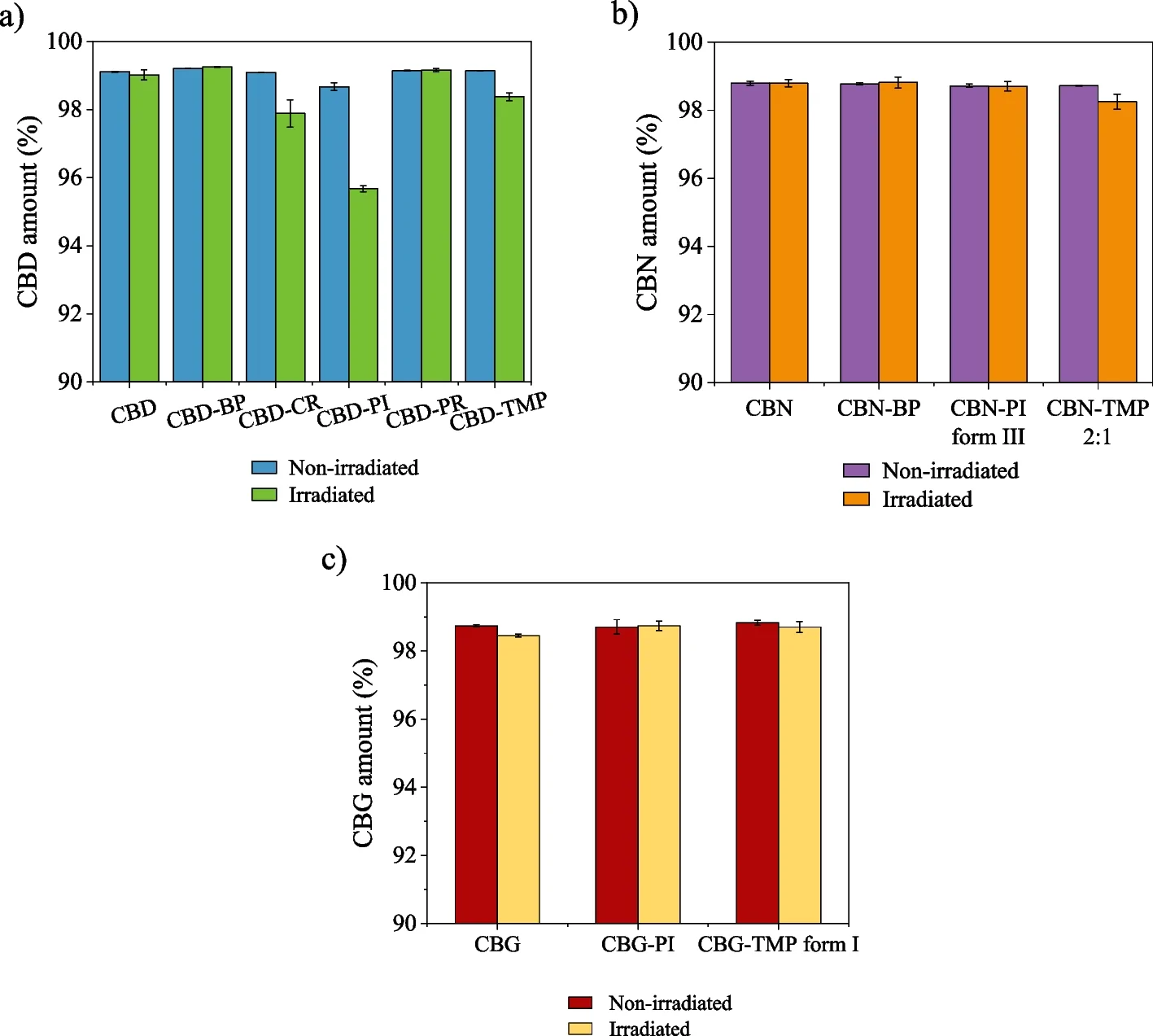
**a** The CBD amount in non-irradiated cocrystals and pure CBD and after exposure to irradiation, **b** the CBN amount in non-irradiated cocrystals and pure CBN and after exposure to irradiation, and **c** the CBG amount in non-irradiated cocrystals and pure CBG and after exposure to irradiation

The irradiated CBD samples (Fig. 9a), namely pure CBD, CBD-BP, and CBD-PR, did not show any decrease in the CBD amount. Their crystalline state was fully preserved, confirmed using XRPD analysis (Figure SI 41a, b, e). A slight colour change from white to light yellow was observed for pure CBD. The sample CBD-CR and CBD-TMP showed a slight decrease.

in the CBD amount, when the experimental error is taken into account, and they changed their colour to pink and beige, respectively. However, the XRPD analysis (Figure SI 41c, f) confirmed that the initial crystalline state of cocrystals was present only. The minor decrease in CBD amount and the colour changes may be attributed to surface oxidation processes within the sample (Urbanczyk-Lipkowska et al. 2011), which are detectable by UPLC, but do not influence the crystalline form, thus they remain invisible in the XRPD patterns. Among the CBD cocrystals, the CBD-PI cocrystal was the most affected by light exposure. The colour was changed to dark brown, and the XRPD analysis (Figure SI 41 d) revealed partial disintegration of the cocrystal, as evidenced by the presence of CBD peaks (e.g., 9.54, 12.42, 17.25° 2*θ*) in the XRPD pattern. The observed decrease in CBD amount is more likely associated with the degradation of the piperazine rather than CBD itself, as pure CBD did not exhibit any significant degradation upon irradiation.

Regarding the CBN samples (Fig. 9b), their stability was not influenced by light radiation. Only a slight decrease in CBN amount was observed for CBN-TMP 2:1 cocrystal, accompanied by a colour change to beige, which can be analogously attributed to surface oxidation processes, similar to those observed for CBD-TMP. A similar observation of colour change to brown was observed for CBN-PI form III. Nevertheless, XRPD analysis (Figure SI 42) did not reveal any crystalline transformation for any of the CBN samples.

Light radiation did not significantly affect the stability of CBG samples (Fig. 9c). Only pure CBG showed a negligible decrease in CBG amount. No colour changes were observed in any of the CBG samples. Furthermore, XRPD analysis (Figure SI 43) confirmed that the crystalline state remained unchanged in both non-irradiated and irradiated samples.

Storage of CBD, CBN, and CBG samples in darkness (non-irradiated) did not lead to any degradation after exposure to the defined light conditions, as expected.

Overall, light exposure had no effect on cocrystals containing 4,4’-bipyridine, CBD-PR, CBG-PI and CBG-TMP form I. The remaining cocrystals exhibited minor colour changes; however, these changes were not connected with significant impurity formation. On the other hand, CBD-PI cocrystal was sensitive to irradiation and underwent a partial disintegration of the cocrystal.

#### Oxidative stability

##### Autooxidation

*CBD and its cocrystals*

**Fig. 10:**
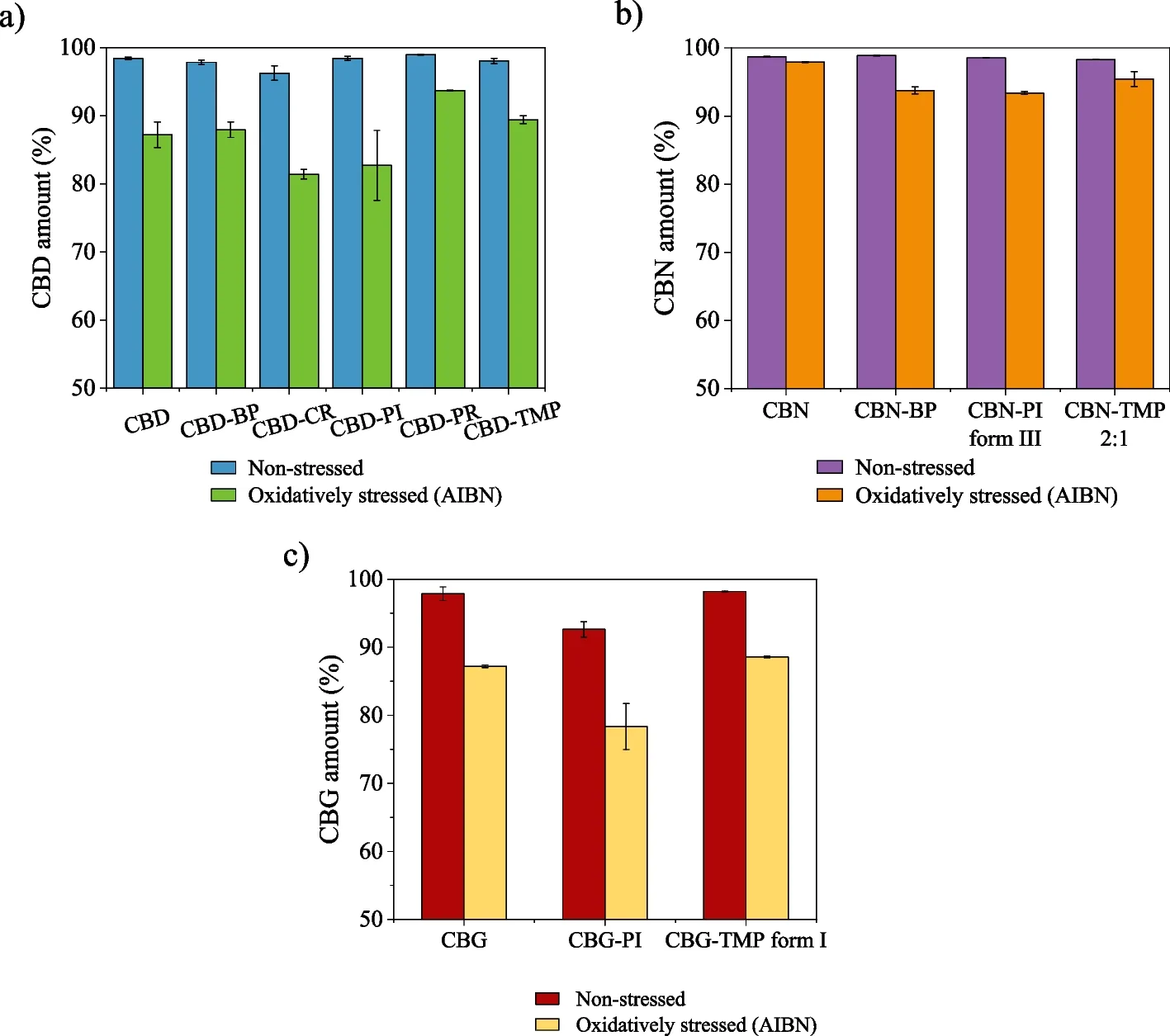
**a** The CBD amount in non-stressed cocrystals and pure CBD, and after exposure to AIBN-induced oxidative stress, **b**) the CBN amount in non-stressed cocrystals and pure CBN, and after exposure to AIBN-induced oxidative stress, and **c**) the CBG amount in non-stressed cocrystals and pure CBG, and after exposure to AIBN-induced oxidative stress

The results of autoxidative stressing experiments are shown in Fig. 10a. Among the CBD cocrystals, CBD-PR exhibited the highest stability against the radical-mediated oxidation and also provided a protective effect to pure CBD. In contrast, the remaining CBD cocrystals exhibited a comparable degradation level.

The sensitivity of pure CBD is attributed to its molecular structure, which contains two hydroxyl groups that can participate in radical initiation and propagation. This behaviour was reported across a wide group of phenolic compounds, which interact with radical species, thereby exhibiting antioxidant properties (Mathew et al. 2015). Furthermore, CBD contains a non-aromatic cyclohexene ring, which can also participate in the radical attack. This sensitivity can be further supported by the morphology (Fig. 1a), which shows hydroxyl groups exposed on the largest facets, which can provide a site for first interaction with radicals.

In the case of CBD-BP, the predicted morphology (Fig. 1b) reveals that the hydroxyl groups of CBD are oriented toward the interior of the crystal, and they are not exposed on the surface. However, the nitrogen atoms of BP are exposed on the major crystal facets. Nitrogen atoms, as an electron-rich region, can facilitate interaction with a free radical, similar to oxygen atoms (Taniguchi 2017). Consequently, the heteroatoms provide oxidative degradation, even though, the CBD hydroxyl groups are shielded.

The CBD-CR cocrystal exhibits the highest degradation among the CBD cocrystals. This behaviour is most likely associated with its high hygroscopicity, which is influenced by the dominant exposure of the hydroxyl group of CR on the crystal surface (Fig. 1c). The increased hygroscopicity promotes moisture uptake and enhances the oxygen availability on the crystal surface, thereby accelerating oxidative degradation.

For CBD-PI and CBD-TMP, the crystal structures are not available. Nevertheless, as both coformers contain nitrogen atoms, it can be assumed that the oxidation is mediated by the heteroatoms exposed at the crystal surfaces.

In contrast, CBD-PR exhibits enhanced oxidative stability. Although the morphology (Fig. 1d) reveals hydroxyl groups of L-proline on the dominant crystal facets, these functional groups are probably sterically shielded by neighbouring L-prolines, which together form square motifs (Koryťáková et al. 2025). This probably suppresses interactions with free radicals and improves the resistance of CBD-PR to autooxidation.

*CBN and its cocrystals*

In Fig. 10b, the results of autoxidative stressing experiments for CBN and its cocrystals are shown. CBN cocrystals exhibit higher oxidative stability compared to CBD cocrystals and pure CBN showed negligible degradation. This enhanced stability compared to CBD can be attributed to the presence of only one hydroxylic group in the CBN structure, which suppresses the radical propagation (Mathew et al. 2015). Moreover, the morphology prediction (Fig. 1e) exhibits lower exposure of hydroxyl groups compared to CBD.

The CBN cocrystals exhibit slightly higher, but relatively similar, levels of degradation compared to pure CBN. The predicted morphology of CBN-BP cocrystal (Fig. 1f) shows nitrogen atoms of the BP exposed on the dominant facets, while the hydroxyl groups of CBN are oriented toward the crystal interior, similar to the CBD-BP cocrystal. The exposed nitrogen atoms likely promote radical reactions and lead to minor degradation.

For CBN-PI form III, the nitrogen atoms of PI are exposed only on smaller facets, whereas the hydroxyl groups of CBN are exposed on the major crystal surfaces (Fig. 1g). In the case of CBN-TMP 2:1, the predicted morphology (Fig. 1h) reveals exposure of heteroatoms from both the coformer and the CBN molecule on the same smaller facets. These exposed hydroxyl groups and heteroatoms can initiate the radical-mediated oxidation.

*CBG and its cocrystals*

The results of the AIBN-induced oxidative stressing are shown in Fig. 10c. Pure CBG and its cocrystals exhibited oxidative instability comparable to that observed for CBD and its cocrystals. This similar behaviour can be attributed to their related chemical structures, as both compounds contain two hydroxyl groups and a carboxylic acid group. In addition, the predicted morphology (Fig. 1i) shows that hydroxylic groups are exposed on the crystal surface, which may facilitate oxidative interactions.

The CBG-PI cocrystal showed higher oxidative instability than pure CBG. The predicted morphology (Fig. 1j) reveals a substantial presence of hydroxyl groups at the crystal surface. Although they are partially shielded, their accessibility may promote radical interaction and increase the cocrystal instability.

In contrast, CBG-TMP form I exhibited oxidative instability similar to that of pure CBG. This observation is consistent with the predicted morphology (Fig. 1k), which shows mostly aliphatic chains exposed at the surface. However, nitrogen atoms from TMP are also present on the crystal surface and may participate in degradation processes, leading to a stability profile comparable to that of pure CBG, in which the hydroxyl groups are exposed on the surface.

##### Peroxide oxidation

*CBD and its cocrystals*

**Fig. 11:**
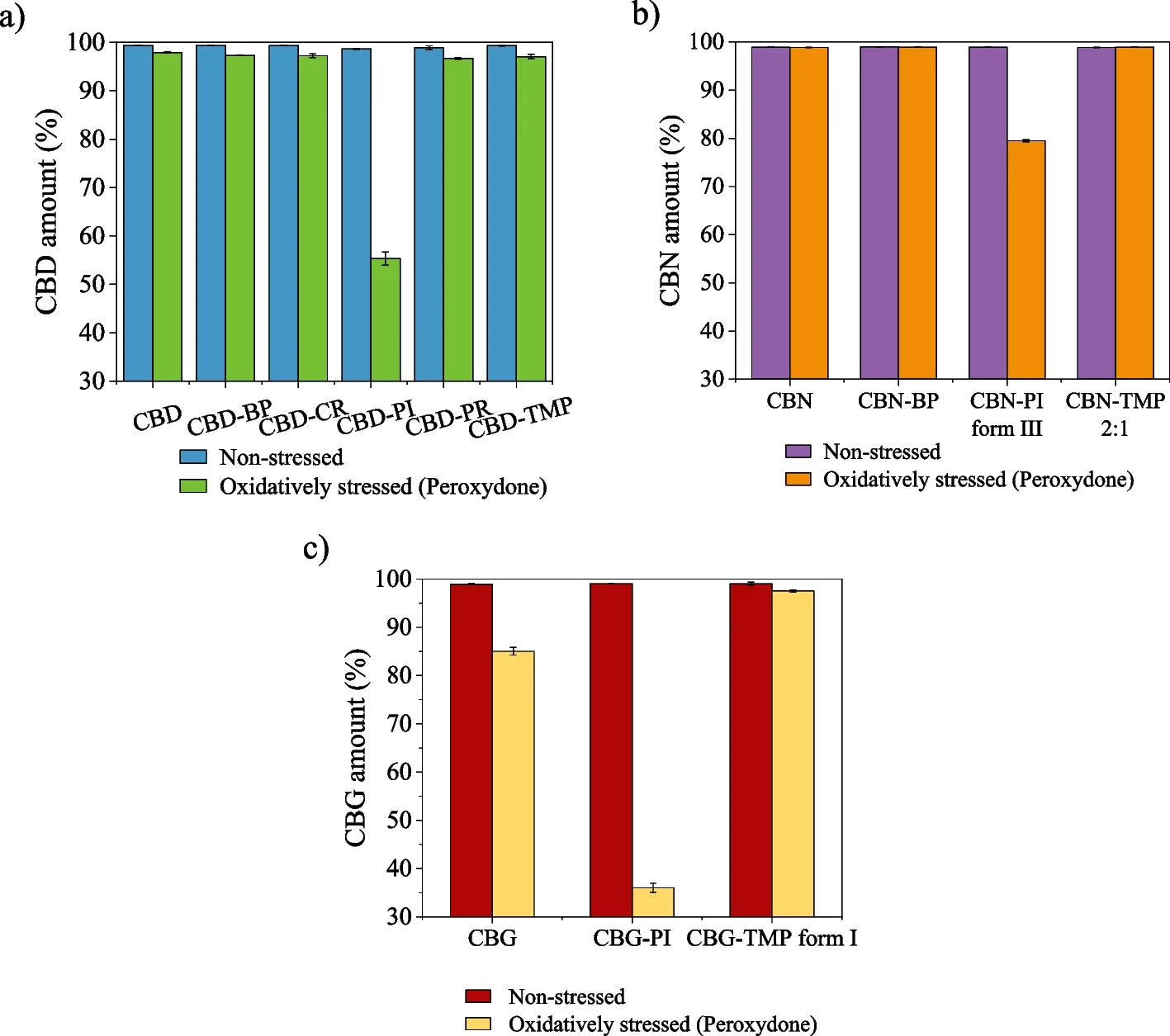
**a** The CBD amount in non-stressed cocrystals and pure CBD and after exposure to peroxide-induced oxidative stress, **b** the CBN amount in non-stressed cocrystals and pure CBN and after exposure to peroxide-induced oxidative stress and **c** the CBG amount in non-stressed cocrystals and pure CBG and after exposure to peroxide-induced oxidative stress

The results of peroxide-mediated oxidation experiments are shown in Fig. 11a. Although the peroxide-mediated oxidation proceeds via the generation of radical species (•OH), these radicals usually exhibit limited propagation compared to those formed during radical-mediated autooxidation, which leads to significantly lower overall degradation (Wasylaschuk et al. 2007). Accordingly, all the experiments resulted in lower degradation of the pure CBD and its cocrystals than radical-induced oxidation.

Pure CBD shows only minimal degradation, which indicates that peroxide-mediated oxidation is less efficient than radical oxidation. Similarly, cocrystals CBD-BP, CBD-CR, CBD-PR, and CBD-TMP exhibit degradation profiles comparable to those of pure CBD. In contrast, CBD-PI showed pronounced instability. Notably, the sample exhibited a colour change to red after removal from the stability chamber. This instability can be attributed to the presence of piperazine, whose secondary amine nitrogen atoms may possess lone electron pairs. These lone pairs can interact with the peroxide and radical intermediates, facilitating cocrystal degradation.

Although L-carnitine and L-proline also contain nitrogen atoms, their electronic states differ from that of piperazine. L-carnitine exists as a quaternary ammonium species, in which the nitrogen atom lacks a lone electron pair. Similarly, the nitrogen atom of L-proline is a secondary amine without a lone electron pair. In the case of 4,4’-bipyridine and tetramethylpyrazine, which exhibit lone electron pairs, the nitrogen atoms are part of aromatic systems, which can probably contribute to structural stabilisation and suppress the degradation and radical propagation.

*CBN and its cocrystals*

In Fig. 11b, the results of peroxide-mediated oxidation results are presented. Pure CBN and its cocrystals, except CBN-PI form III, exhibit a high resistance toward peroxide-mediated oxidation, and remain stable.

In contrast, CBN-PI form III exhibits pronounced degradation, which is consistent with the behaviour observed for the CBD-PI cocrystal. The enhanced degradation is attributed to the presence of nitrogen atoms with lone-pair electrons, which can facilitate interaction with peroxide radicals and thereby decrease the stability of the cocrystal.

*CBG and its cocrystals*

The peroxide-mediated oxidation results for CBG and its cocrystals are presented in Fig. 11c. In contrast to pure CBD and CBN, CBG exhibited pronounced instability upon exposure to the peroxide-mediated stressing agent. This increased degradation is likely related to the presence of accessible double bonds in the CBG side chain, which may act as reactive sites for the peroxide-mediated oxidation. Similarly, CBG-PI showed the greatest degradation, among all studied systems. This pronounced instability likely arises from the combined contributions of the reactive CBG molecule and the piperazine coformer, whose nitrogen atoms contain lone electron pairs, facilitating interaction with peroxide radicals. A comparable behaviour was previously observed for CBD-PI and CBN-PI form III. Additionally, the CBG-PI sample changed its colour to bright reddish after removal from stability chambers, which further confirms the observed oxidative instability.

In contrast, CBG-TMP form I exhibited greater stability than pure CBG. In this case, the coformer probably provides a stabilizing effect for CBG, through its aromatic structure, which could contribute to electron delocalization and thereby suppress the peroxide-mediated oxidation.

##### Summary of Oxidative Studies

In conclusion, the CBD and its cocrystals showed pronounced degradation toward autooxidation, except for CBD-PR, which exhibited improved stability. CBG and its cocrystals showed degradation profiles comparable to those of CBD systems. In contrast, pure CBN exhibited high stability, with almost no detectable degradation, and its cocrystals were less affected than the CBD and CBG cocrystals. We observed that autooxidation depends on the presence of heteroatom-containing functional groups in the chemical structures of the studied systems. Furthermore, it is strongly dependent on the crystal morphology and the exposed heteroatoms on crystal surfaces, which can initiate and propagate radical reactions, thereby accelerating degradation.

Under peroxide-mediated oxidation, both CBD and its cocrystals showed lower degradation than under radical-induced oxidation. Only a minor decrease in CBD amount was observed for the cocrystals, except CBD-PI, which showed the highest degradation among the cocrystals. A similar behaviour was observed for CBN-PI form III, while pure CBN and the rest of the cocrystals remained stable. In contrast, CBG and CBG-PI showed pronounced degradation, likely due to the presence of accessible double bonds in their structures, which may serve as reactive sites for peroxide-mediated oxidation. Notably, CBG-TMP form I exhibited improved stability, highlighting the protective effect of the coformer on pure CBG. The instability of cocrystals containing piperazine may be influenced by the coformer, whose nitrogen atoms possess free lone electron pairs, which may facilitate interaction with peroxide species and thereby lower the cocrystal stability. Therefore, we can assume that peroxide-mediated degradation depends on the nature of the cocrystals, which may reduce the API's stability.

Overall, both CBD and CBN cocrystals were less affected by peroxide-mediated oxidation than by radical-mediated oxidation, highlighting distinct mechanisms under these stress conditions. CBD and CBG cocrystals exhibited higher oxidative instability than CBN cocrystals, which is connected to the higher stability of CBN. The enhanced stability can be attributed to its higher melting point and to structural features, specifically the number of hydroxylic groups. This observation is consistent with a previous study on the antioxidation activity of cannabinoids, which found that CBD and CBG showed higher antioxidant activity than CBN (Dawidowicz et al. 2021).

## Conclusion

In this study, we investigated the stability of cannabinoid cocrystals under a range of solid-state stress conditions, including thermal, pH-dependent, photostability, and oxidative degradation.

The thermal stability experiments showed that CBN-BP and CBG-PI exhibited greater stability than the pure APIs. Overall, the thermal stability of the cocrystals appears to be governed by multiple factors, including melting point, interaction energy, and the chemical nature of the coformer. Furthermore, we could observe similar trends in stability behaviour for CBD and CBN cocrystals, primarily depending on the used coformer, whereas CBG cocrystal stability depended on the melting point.

The pH-dependent stability studies revealed trends similar to those observed for the cocrystals compared to their corresponding pure APIs. Interestingly, the majority of the cocrystals showed lower API loss rates at the lowest and highest pH values, whereas intermediate pH values were associated with the highest API loss rates. Notably, the cocrystal with 4,4’-bipyridine exhibited negligible API loss at pH 9.7, suggesting a protective effect of the coformer.

Photostability experiments showed minimal impurity formation for most samples, and only slight changes in colour were sometimes observed, which could be attributed to oxidative processes occurring on the crystal surfaces.

Oxidative studies revealed that radical-mediated oxidation had a greater impact on the cocrystal stability than peroxide-mediated oxidation. Furthermore, the results show that the radical-mediated oxidation is strongly influenced by the exposure of heteroatoms on crystal surfaces, which can facilitate radical initiation and propagation. In contrast, peroxide-mediated oxidation is less dependent on the surface morphology and is primarily influenced by the chemical nature of the coformer.

Overall, the results revealed distinct stability trends across the investigated coformers. Cocrystals containing 4,4’-bipyridine and CBD-PR demonstrated a clear stabilizing influence on the cannabinoids, whereas cocrystals with piperazine and CBD-CR were most susceptible to instability. The observed stability behaviour indicates that cocrystallization can either enhance or reduce stability depending on structural factors such as crystal packing, coformer properties, and the accessibility of reactive functional groups within the crystal structure. These findings highlight the importance of crystal structure analysis and coformer selection in determining the stability of cannabinoid cocrystals. Furthermore, they may support the rational design of more stable cannabinoid solid forms for future pharmaceutical development.

## Supplementary Information

Supplementary Material 1.

## Data Availability

All data will be deposited to Zenodo repositary.
