# Supplementary material for: Structure–stability relationships of pure cannabinoids and their cocrystals under forced degradation conditions
Source: J Cannabis Res. 2026 Jul 21;8:109. doi: 10.1186/s42238-026-00474-0 (PMC13673648; doi:10.1186/s42238-026-00474-0)
Supplement: Supplementary file 1 [file 42238_2026_474_MOESM1_ESM.docx]

**Structure–Stability Relationships of Pure Cannabinoids and Their Cocrystals under Forced Degradation Conditions**

Supplementary Information

*Adéla Koryťáková^1^, Argyro Chatziadi^1^, Jakub Heřt^3^, Jan Rohlíček^2^, Eliška Zmeškalová^1,2^, Josef Beránek^3^, Miroslav Šoóš^1,*^*

^1^Department of Chemical Engineering, University of Chemistry and Technology in Prague, Technická 3, 16628, Prague 6, Czech Republic

^2^Institute of Physics of the Czech Academy of Sciences, Na Slovance 2, 182 00, Prague 8, Czech Republic

^3^Zentiva k.s., U Kabelovny 130, 10237, Prague 10, Czech Republic

*corresponding author

# SI 1 Screening and Cocrystal Preparation

## 1.1 Materials

The coformers (namely, 2,2′-bipyridine; 4,4′-bipyridine; 4-methylpyridin-N-oxide; L-arginine; betaine; caffeine; L-carnitine; L-cysteine; L-glutamic acid; L-glutamine; L-glycine; hippuric acid; indole; isonicotinic acid N-oxide; isonicotinamide; lidocaine; L-lysine; nicotinamide;
L-phenylalanine; piperazine; polydatin; L-proline; pyridine-N-oxide; quercetin; riboflavin; tetramethylpyrazine; L-tryptophan; L-tyrosine; L-valine) were purchased from Sigma-Aldrich (St. Louis, USA) and were used as received. All coformers have purity higher than 98 %.

Solvents such as butyl acetate (BA), ethanol (EtOH), heptane (HP), methanol (MeOH) and propan-2-ol (PrOH) were purchased from PENTA (Prague, Czech Republic).

## 1.2 Screening Experiments and Characterization

The screening of CBN cocrystals was performed in a ratio 1:1 for all initial experiments. A total 29 coformers were selected based on functional-group complementarity, molecular size and structural information from the Cambridge Structural Database (CSD). The CSD^1^ was used as a source of structural information for a qualitative synthon-based analysis. The molecular structure of CBN was first inspected to identify functional groups capable of strong and directional hydrogen bonding. The hydroxyl group was identified as the most promising interaction site. ConQuest^2^ was then used to search the CSD for multicomponent organic structures containing alcohol/hydroxyl groups, in order to identify common heterosynthons formed by non-solvent alcohols with non-solvent coformers. This analysis highlighted several relevant coformer classes, including pyridine and pyridine-N-oxide derivatives, amides, cyclic or aromatic nitrogen-containing compounds, and zwitterionic molecules. Acid–base compatibility was considered only qualitatively, since proton transfer from the hydroxyl group of CBN to the selected coformers was not expected.

In addition, we included three coformers, namely betaine, L-proline and tetramethylpyrazine, for which CBN cocrystals are reported in the patent literature.^3, 4^ For these patented cocrystals, the reported cocrystal stoichiometries were also investigated.

### 1.2.1  Slurry Experiments

Each slurry was prepared by suspending 20 mg of CBN and an equimolar amount of the corresponding coformer in approximately 0.25 mL of either a heptane/ethanol mixture (ratio 95:5), propanol or butyl acetate in a 2 mL vial. The slurry experiments were carried out for 7 days at 25 °C and 750 rpm in a thermomixer. The obtained material was filtered and dried.

### 1.2.2 Milling Experiments

Milling experiments were performed with a Retsch MM500 mixer mill. Approximately 50 mg of CBN and an equimolar amount of the coformer were mixed in a 2 ml polypropylene milling jar with two 5 mm stainless steel balls. A liquid additive (MeOH or HP) was added in an amount of 5 μl into the plastic jars which were immediately closed to prevent evaporation of the liquid additive. The grinding experiments were carried out at a frequency of 25 Hz for 20 minutes.

CBN-tetramethylpyrazine (1:1) form II was prepared using low temperature neat grinding (5 °C), a frequency of 30 Hz and a milling time of 90 minutes.

## 1.3 Results

### 1.3.1 Screening Experiments

The screening experiments resulted in four cocrystals namely CBN-4,4’-bipyridine (BP), CBN-piperazine (PI) form I (2:1), CBN-tetramethylpyrazine (TMP) form I and CBN-TMP in a 2:1 ratio out of 29 tried coformers. All the obtained phases were characterized using XRPD to confirm successful cocrystal formation. Slurry experiments provided access to cocrystals CBN-BP and CBN-PI form I. For CBN-PI form I, although the initial experiment was performed using a 1:1 ratio, the obtained phase corresponded to a 2:1 stoichiometry with excess of piperazine. Therefore, the CBN-PI form I cocrystal was further prepared using a 2:1 ratio to obtain a pure phase. Through milling, we were able to obtain not only these two phases, but also both cocrystals with TMP mentioned in the patent literature^3^: CBN-TMP in a 1:1 ratio (form I) and CBN-TMP in a 2:1 ratio. The last obtained cocrystal, CBN-TMP form II in a 1:1 ratio, was obtained unexpectedly during attempts to prepare a coamorphous phase. The milling experiments also provided access to three polymorphs of the CBN-PI cocrystal, one of which later was proved to be a disappearing polymorph.^5^ Overall, milling experiments showed a higher success rate and enabled the formation of additional phase compared to solution-based methods.

A comparison of all CBN cocrystals, except CBN-PI and its polymorphs, obtained during the screening with the starting material is shown in Figure SI 1. For each phase, the crystal structure was successfully solved, thus the simulated XRPD patterns are included to confirm the phase purity. However, the two additional patented cocrystals^3, 4^ with betaine and L-proline were not within the positive results of the screening, despite testing multiple conditions including solvents and various time of milling.

b)

a)

Figure SI 1. a) Experimental and calculated XRPD patterns of CBN–BP and CBN–TMP (2:1) cocrystals compared with their starting materials and b) experimental and calculated XRPD patterns of CBN-TMP (1:1) polymorphs I and II compared with their starting materials.

# SI 2 Characterization of CBN Cocrystals

The additional characterization of the newly prepared phases (namely, CBN-BP, CBN-TMP (1:1) form I, CBN-TMP (1:1) form II and CBN-TMP (2:1)), including detailed crystal structure description, interaction energy calculations, thermal behaviour and intrinsic dissolution rate are presented in this section. The additional characterization of the cocrystal CBN-PI and its polymorphs was published previously.^6^

## 2.1 Crystal Structure and Morphology of CBN and Its Cocrystals

### 2.1.1 Single Crystal and Powder X-ray Diffraction Measurement

#### 2.1.1.1 Sample Preparation

The single crystal of CBN-BP was prepared by combining CBN and 4,4′-bipyridine in 1,4-dioxane, CBN-TMP form I in a 1:1 ratio was prepared in a mixture of ethyl acetate and heptane in a ratio 1:10, while CBN-TMP in a 2:1 ratio was prepared in heptane. These mixtures were left to evaporate slowly until the single crystals were formed. The mixtures were heated to 35 °C to dissolve the powder and then allowed to cool down, followed by slow evaporation until the single crystals were formed. The preparation of the single crystals did not present any challenges in contrast to the CBN-TMP form II in a 1:1 ratio. Because the CBN-TMP form II can only be obtained by low temperature milling, our attempts to grow single crystals by slow evaporation or seeding resulted in the formation of CBN-TMP form I instead. Therefore, we employed XRPD to solve the crystal structure from powder data.

#### 2.1.1.2 Measurement

Single crystal X-ray diffraction (SCXRD) measurement was performed using two four-circle CCD Rigaku diffractometers: Xcalibur Gemini ultra diffractometer using the Cu-Kα radiation (λ = 1.54184 Å) from a fine-focus sealed X-ray tube with a graphite monochromator and Atlas S2 CCD detector (CBN-BP), and XtaLAB Synergy R, equipped with a rotating anode Cu X-ray tube and a HyPix-Arc 150 detector (CBN-TMP 1:1 and 2:1). Both diffractometers use a Cryojet chiller. The data reduction and absorption correction were done with CrysAlisPro software.^7^ The structures were solved by charge flipping methods using Superflip software and refined by full matrix least squares on squared value using Crystals (ref.^8^) and Jana2020 software (ref.^9^). MCE software^10^ was used for the visualization of residual electron density maps. All H atoms were placed from the residual electron density map, and the C-H were constrained to ideal geometries.

Sample CBN-TMP (1:1) form II was ground and placed in the 0.5 mm borosilicate-glass capillary. Powder diffraction data were collected using the Debye-Scherrer transmission configuration on the powder diffractometer Empyrean of PANalytical (Cu, Kα1 = 1.54184 Å) that was equipped with a focusing mirror, a capillary holder and a PIXcel^3D^ detector. The data were collected from 3° to 80° 2*θ*, with a step of 0.013° and with an overall 20h measurement time.

The structures were deposited into the Cambridge Structural Database under numbers 2512838 (CBN-BP), 2512839 (CBN-TMP 1:1 form I), 2512840 (CBN-TMP 1:1 form II) and 2512841 (CBN-TMP 2:1). Details of crystal structure solutions are in Table SI 1.

Table SI 1. Crystallographic data and details of refinement of CBN cocrystals

|  | **CBN-BP** | **CBN-TMP (1:1) form I** | **CBN-TMP (1:1) form II** | **CBN-TMP 2:1** |
| --- | --- | --- | --- | --- |
| Sample | Single crystal | Single crystal | Powder | Single crystal |
| Empirical formula | C_31_H_34_N_2_O_2_ | C_29_H_38_N_2_O_2_ | C_29_H_38_N_2_O_2_ | C_50_H_64_N_2_O_4_ |
| Formula weight | 466.62 | 446.63 | 446.6 | 757.07 |
| Crystal system | Monoclinic | Monoclinic | Monoclinic | Monoclinic |
| Space group | *P*2_1_/*n* | *P*2_1_/*n* | *P*2_1_/*c* | *P*2_1_/*c* |
| T (K) | 120 | 90 | 293 | 95 |
| Radiation | Cu *K*α | Cu *K*α | Cu *K*α1 | Cu *K*α |
| a (Å) | 8.6506 (1) | 14.8951 (5) | 11.0569 (2) | 18.3384 (7) |
| b (Å) | 18.5056 (1) | 10.1066 (3) | 11.5714 (3) | 16.2999 (6) |
| c (Å) | 16.1684 (1) | 17.5453 (5) | 20.9805 (4) | 14.4339 (5) |
| α (°) | 90 | 90 | 90 | 90 |
| β (°) | 97.0399 (7) | 104.399 (3) | 102.1504 (10) | 93.462 (3) |
| γ (°) | 90 | 90 | 90 | 90 |
| V (Å^3^) | 2568.80 (4) | 2558.28 (14) | 2624.19 (10) | 4306.6 (3) |
| Z | 4 | 4 | 4 | 4 |
| Reflns. collected | 47060 | 17813 | - | 29636 |
| Indep. reflns | 4671 | 4985 | - | 8124 |
| GOF | 0.9764 | 0.9860 | 2.9802 | 0.9794 |
| R_1_, wR_2_ [I > 2δ(I)] | 0.0342, 0.0861 | 0.0441, 0.1070 | 0.0553, 0.0518 | 0.0750, 0.1747 |
| R_1_, wR_2_ (all data) | 0.0365, 0.0883 | 0.0577, 0.1136 | 0.0585, 0.0522 | 0.0750, 0.1747 |
| Δρ_max_, Δρ_min_  (e Å^−3^) | 0.21, −0.19 | 0.33, −0.25 | 0.10, −0.15 | 0.57, −0.26 |
| R_p_, R_wp_, R_exp_ | - | - | 0.023, 0.032, 0.011 | - |
| CCDC number | 2512838 | 2512839 | 2512840 | 2512841 |

The crystal structure of CBN-TMP 1:1 was solved from high-resolution laboratory XRPD data using the direct-space approach as implemented in the program FOX (ref.^11^), with the unit cell parameters determined by Dicvol (ref.^12^) within FOX. The sample contained a small amount of impurity, which was identified as CBN-TMP 2:1. The presence of the impurity was neglected during refinement because its Bragg peaks had very low intensity. The initial model of CBN was taken from the known crystal structure available in the CSD under the refcode *CANNOL01* (ref.^13^). The tetramethylpyrazine molecule was built using ChemSketch (ref.^14^). Rietveld refinements of the initial model was performed with Jana2020 (ref.^9^), where the structure was refined using bond-length and bond-angle restraints consistent with the initial molecular model. Bond distances and bond angles were restrained to target values with standard uncertainties of 0.001 Å and 0.01°, respectively. Hydrogen atoms were placed in geometrically calculated positions. In the final stage of refinement, all atomic positions, three isotropic ADPs (one for tetramethylpyrazine and two for CBN - one assigned to the pentyl chain and the other to the remainder of the molecule), as well as the profile parameters, were refined. The resulting profile fit is shown in Figure SI 2.

Figure SI 2. The final Rietveld fits of XRPD pattern of CBN-TMP (1:1) form II. Black dots are measured data, red is the calculated profile, blue is the difference between them and green bars are Bragg’s positions.

#### 2.1.1.3 Interaction Energies Calculations

Interaction energies and energy frameworks were determined using CrystalExplorer17 software (version 17.5, revision f4e298a)^15^. Molecular wave functions were generated with the built-in Tonto module at the “accurate” setting, which is using the B3LYP/6-31G(d,p) level of theory. Visualizations of the energy frameworks were created using the same software.

### 2.1.2 Results

To gain deeper insight into the CBN cocrystals, their crystal structures and interactions within the crystal lattice were analysed. Furthermore, we successfully solved all the crystal structures of the prepared phases. In addition, the morphology was predicted to identify the functional groups exposed at the crystal surfaces of each cocrystal. Full crystallographic data and details of the structure refinement are provided in Section SI 1.

##### CBN

The crystal structure of pure CBN has been described in detail in our previous publication.^6^ The predicted morphology (Figure SI 3) indicates that on the crystal surfaces are mostly hydrophobic groups exposed, which contributes to the overall hydrophobic character of the CBN.

Figure SI 3. Predicted morphology of CBN.

##### CBN-BP

The CBN-BP cocrystal crystallizes in the monoclinic system with a space group P2_1_/n. The asymmetric unit (Figure SI 4a) contains one molecule of CBN and 4,4′-bipyridine each, while the unit cell contains four of each. The hydrogen-bond system (Figure SI 4a) is simple, with CBN and BP molecules bonded through a single interaction, OH⋯N(Ar). The strongest interaction energy (Figure SI 4b) is approximately −43.3 kJ mol^−1^ and it occurs between the CBN and 4,4′-bipyridine molecules, while the CBN-CBN interaction is lower, around −34.3 kJ mol^−1^. According to the morphology prediction (Figure SI 4c), the major faces mainly present the aliphatic CBN chain, whereas the BP molecules are located on the smaller faces. As a result, these functional groups give the cocrystal a mainly hydrophobic surface character.

Figure SI 4. Crystal structure of CBN-BP of a) asymmetric unit, b) calculated interaction energy, c) predicted morphology.

##### CBN-PI form III

The crystallographic information of CBN-PI form III, identified as the most stable polymorph, has been published previously.^6^ The predicted morphology (Figure SI 5) reveals that the crystal surface exhibits mostly with hydrophobic parts (aromatic ring), resulting in an overall hydrophobic surface.

Figure SI 5. Predicted morphology of CBN-PI form III.

##### CBN-TMP (1:1) form I

CBN-TMP (1:1) form I has a monoclinic system with a space group P2_1_/n. Its asymmetric unit (Figure SI 6a) consists of one molecule of CBN and one molecule of TMP. The unit comprises four molecules of each kind. The CBN and TMP molecules are connected through an OH⋯N(Ar) hydrogen bond (Figure SI 6a). The TMP molecules are located in the channel formed by the CBN molecules. The strongest interaction (−53.4 kJ mol^−1^, Figure SI 6b) occurs between the molecules of TMP and CBN involved in the hydrogen bond. The predicted morphology (Figure SI 6c) reveals both hydrophobic (aromatic ring) and hydrophilic (CBN hydroxyl group) groups on the crystal surface of the cocrystal. Nevertheless, the hydrophobic part shown on the crystal surface is dominant as it is present on the largest facets.

Figure SI 6. Crystal structure of CBN-TMP (1:1) form I of a) asymmetric unit, b) calculated interaction energy, c) predicted morphology.

##### CBN-TMP (1:1) form II

CBN-TMP (1:1) form II crystallizes in the monoclinic system in the space group P2_1_/c.
In the asymmetric unit (Figure SI 7a), there is one molecule of CBN and one tetramethylpyrazine molecule. The unit cell comprises of four molecules of CBN and four molecules of TMP. The two molecules in the asymmetric unit are connected through an OH⋯N(Ar) hydrogen bond (Figure SI 7a). The crystal arrangement of CBN creates cavities that accommodate the TMP molecules. The strongest calculated interaction energy between CBN and TMP (Figure SI 7b) involves the mentioned hydrogen bond, with a value of −48.1 kJ mol^−1^. The predicted morphology (Figure SI 7c) shows hydrophobic (aromatic ring and aliphatic tail) and hydrophilic (CBN hydroxyl group) groups on the crystal surface. However, the hydrophobic groups are exposed on the dominant facets, as observed for CBN-TMP (1:1) form I.

Figure SI 7. Crystal structure of CBN-TMP (1:1) form II of a) asymmetric unit, b) calculated interaction energy, c) predicted morphology.

##### CBN-TMP (2:1)

CBN-TMP (1:1) form II crystallizes in the monoclinic system in the space group P2_1_/c.
In the asymmetric unit (Figure SI 8a), there is one molecule of CBN and one tetramethylpyrazine molecule. The unit cell comprises of four molecules of CBN and four molecules of TMP. The two molecules in the asymmetric unit are connected through an OH⋯N(Ar) hydrogen bond (Figure SI 8a). The crystal arrangement of CBN creates cavities that accommodate the TMP molecules. The strongest calculated interaction energy between CBN and TMP (Figure SI 8b) involves the mentioned hydrogen bond, with a value of −54.0 kJ mol^−1^. The predicted morphology (Figure SI 8c) shows hydrophobic (aromatic ring and aliphatic tail) and hydrophilic (CBN hydroxyl group) groups on the crystal surface. However, the hydrophobic groups are exposed on the dominant facets, as observed for CBN-TMP (1:1) form I.

Figure SI 8. Crystal structure of CBN-TMP (2:1) of a) asymmetric unit, b) calculated interaction energy, c) predicted morphology.

## 2.2 Solution and Solid-state Nuclear Magnetic Resonance (NMR)

### 2.2.1 Measurement

Solution NMR was used to determine the stoichiometry and purity of the prepared materials. The samples were dissolved in *d*_6_-DMSO and the ^1^H NMR spectra were measured by Avance III 500 MHz NMR spectrometer (Bruker, USA) equipped with a Prodigy probe and with a repetition delay of 10 s.

To confirm the cocrystal creation and purity of the obtained material the solid-state NMR was used. The ^13^C NMR spectra were measured by Avance III 400 MHz NMR spectrometer (Bruker, USA) equipped with a 4 mm probe and with 13 kHz spinning.

### 2.2.2 Results

The molar ratios of the prepared cocrystals obtained by milling experiments were confirmed using ^1^H NMR spectroscopy. The analysis confirmed a 1:1 for all investigated cocrystals, except for CBN-TMP (2:1), which showed a 2:1 molar ratio. The ssNMR further confirmed the cocrystal formation through the appearance of distinct chemical shift differences, which represent a unique cocrystal or polymorph. The ssNMR spectra are presented in Figure SI 9.

a)

b)

c)

d)

Figure SI 9. Solid-state NMR spectra of cocrystals a) CBN-BP, b) CBN-TMP (1:1) form I, c) CBN-TMP (1:1) form II and d) CBN-TMP (2:1).

## 2.3 Thermal Analysis

### 2.3.1 Measurement

Thermal behaviour was studied using differential scanning calorimetry DSC 3+ (Mettler Toledo, Switzerland) and thermogravimetric analysis (TGA). For DSC measurements, the aluminium pan was filled with 2 mg of sample. The aluminium pan was sealed and pierced to allow possible solvent vapour to escape and prevent an explosion. The samples were heated from 20 °C to the specific degradation temperature of each sample at a heating rate of 10 °C/min. For TGA experiments, the pan was filled with approx. 2 mg of sample and heated from 30 °C to 300 °C with a heating rate of 5 °C/min. All measurements were carried out in the inert gas atmosphere.

### 2.3.2 Results

The thermodynamical properties of the prepared cocrystals were investigated using TGA and DSC analysis. The TGA and DSC thermograms are shown in Figure SI 10. The TGA and DSC curves of the starting material compared to the cocrystals is shown in Figure SI 11 and Figure SI 12. From the TGA curves (Figure SI 10a), weight losses of approximately 29 % (calculated coformer content 30.5 %) were observed for CBN-TMP (1:1) form I, 30 % (calculated coformer content 30.5 %) for CBN-TMP (1:1) form II and 19 % (calculated coformer content 18 %) for CBN-TMP 2:1. The first weight loss events end at temperatures near 150 °C for CBN-TMP (1:1) polymorphs, while for CBN-TMP 2:1 near 160 °C. The close agreement between experimental and theoretical mass losses indicates that the first thermal event corresponds to the loss of the coformer from the crystal lattice. Similar behaviour has been reported for cocrystals containing relatively volatile coformers, where the initial mass loss is attributed to coformer loss upon heating.^5, 16^

In contrast, no significant weight loss was observed for CBN-BP prior to melting. This observation is consistent with the thermal behaviour of the pure coformers (Figure SI 11). While TMP exhibits the onset of mass loss even at room temperature, BP remains thermally stable up to approximately 80 °C. These results indicate that the intrinsic thermal properties and volatility of the coformer play an important role in determining the thermal behaviour of the corresponding cocrystals.

The DSC thermograms (Figure SI 10b) show a single sharp endotherm for each prepared cocrystal and pure CBN, which indicates their phase purity. The melting point of CBN is at 76 °C (*T*_onset_ = 74 °C). Except CBN-TMP (1:1) form I, the newly prepared cocrystals showed higher melting points than pure CBN. CBN-BP melts at 141 °C (*T*_onset_ = 137 °C), CBN-TMP (1:1) form I at 74 °C (*T*_onset_ = 70 °C), CBN-TMP (1:1) form II at 77 °C (*T*_onset_ = 73 °C) and CBN-TMP 2:1 at 78 °C (*T*_onset_ = 74 °C). The cocrystals with TMP exhibit melting points similar to that of pure CBN. Only CBN-BP showed a significant increase in thermal stability with a remarkable 85 % increase compared to pure CBN.

b)

a)

Figure SI 10. a) TGA thermograms and b) DSC thermograms of CBN and the cocrystals
CBN-BP, CBN-TMP (1:1) form I, CBN-TMP (1:1) form II, CBN-TMP 2:1.

When comparing the melting points of the solid forms in this study, it becomes evident that a higher melting point does not directly correlate with a stronger interaction energy within the crystal lattice. For example, CBN-BP exhibits the highest melting point among the cocrystals; however, it shows the lowest interaction energy (−43.3 kJ mol^−1^). In contrast, CBN-TMP (1:1) form I shows the lowest melting point, even lower than pure CBN, while it shows the third highest interaction energy (−53.4 kJ mol^−1^) among the cocrystals. These observations indicate that thermal stability cannot be predicted solely from interaction energies. Similar behaviour was previously observed for CBD cocrystals^5^, suggesting that thermal stability is governed by multiple factors, including lattice arrangement, symmetry, hygroscopicity, and intermolecular interactions.^17^

a)

b)

c)

d)

Figure SI 11. TGA curves of cocrystals a) CBN-BP, b) CBN-TMP (1:1) form I,
c) CBN-TMP (1:1) form II and d) CBN-TMP (2:1) compared to their starting materials.

a)

b)

c)

d)

Figure SI 12. DSC curves of cocrystals a) CBN-BP, b) CBN-TMP (1:1) form I,
c) CBN-TMP (1:1) form II and d) CBN-TMP (2:1) compared to their starting materials.

## 2.4 Intrinsic Dissolution Rate

### 2.4.1 Measurement

#### 2.4.1.1 Preparation of Samples

The intrinsic dissolution rate was measured using Sirius inForm (Pion Inc., USA) connected to offline High Performance Liquid Chromatography (HPLC) detection. The discs with a diameter of 3 mm, filled with ca. 20 mg of sample, were compressed with a constant load of 100 kg, relaxed for one minute and compressed again with approximately 100 kg for the second minute. The intrinsic dissolution rate was measured in 40 ml of 0.01 M hydrochloric acid buffer (pH = 2.0) containing 0.5 % Tween^®^ 20 at 100 rpm. The samples for HPLC analysis were collected every two minutes, with a volume of 800 μl. After each sampling, the amount taken was immediately replaced with fresh dissolution medium to maintain a constant volume. Each sample was measured in triplicate. IDR was calculated using a linear fit of the 40 minutes of the measurement. The first six minutes were excluded from the analysis because they represent the initial dissolution of the free, uncompressed powder from the disc surface created during preparation. After each measurement, the discs underwent XRPD analysis to confirm the crystal structure of all samples and to rule out any potential polymorphic changes.

#### 2.4.1.2 HPLC Analysis

The HPLC analysis was performed using Waters Alliance e2695 Separation Module (Waters Corporation, USA) with a high-pressure pump, an autosampler, a thermostat and a photodiode-array detector. A Waters XBridge C18 column (Waters Corporation, USA) with dimension of 50 mm length and 4.6 mm internal diameter with particle size of 3.5 μm was used
for the analysis of the released API. The autosampler temperature was set to 25 °C, the column heater was set up at 40 °C during analysis. Ultrapure water with addition of formic acid (0.1 %) and acetonitrile was used as component A and B, respectively of the mobile phase for the separation. The gradient elution was set as follows: *t* (min)/*%* B: 0/30; 1.5/30; 6.5/80; 9/80; 9.5/30; 12/30 with the flow rate of the mobile phase 0.8 ml min^−1^. The injection volume was set to 50 μl per sample. To evaluate the released amount of API, the absorbance at 210 nm was used. The chromatographic data were evaluated using the software Empower™.

### 2.4.2 Results

The IDR measurements were carried out for pure CBN and its cocrystals. However, the 1:1 cocrystals with TMP were not included in the IDR study as they showed pronounced instability under ambient conditions. The IDR results are shown in Figure SI 13. The IDR values are listed in Table SI 2 and the dissolution rate profiles for all prepared CBN cocrystals and pure CBN are shown in Figure SI 14. Each CBN cocrystal showed a lower IDR value than pure CBN (10.56 ± 0.95 μg min^−1^ cm^−2^).

To investigate further the dissolution mechanism, XRPD analysis (Figure SI 15) of the discs after dissolution measurements was performed. In case of CBN-BP and CBN-TMP 2:1 cocrystals, the diffraction patterns confirmed that the cocrystal phases were present after exposure to the dissolution medium, without any solid-state transformation. In contrast, the XRPD analysis of CBN-PI form III showed peaks corresponding to the initial cocrystal phase together with the pure CBN, which indicates partial dissociation of the cocrystal after exposure to the water environment. These solid-state changes are in accordance with the IDR results. In case of CBN-PI form III, dissociation of the cocrystal leads to free CBN molecules, which result in a higher apparent dissolution rate compared to the other cocrystals and comparable to the IDR value of pure CBN. In contrast, for the CBN-BP and CBN-TMP 2:1 cocrystals, where no dissociation of the cocrystal was observed, the availability of free CBN is limited by the cocrystal stability and hydrophobicity, resulting to a slower IDR compared to pure CBN.

Figure SI 13. Comparison of intrinsic dissolution rates for pure CBN and its cocrystals.

The dissolution behaviour is strongly influenced by the physicochemical properties
of the coformers, such their basicity/acidity, as well as the intermolecular interactions and crystal packing within the crystal lattice. Although, CBN-PI form III exhibits the highest interaction energy (−69.4 kJ mol^−1^), piperazine is the most basic coformer among the used coformers.^18^ Under acidic conditions (pH = 2), piperazine is double protonated, which increases its aqueous solubility and enhances its interaction with the aqueous environment.^19^ In the cocrystal, crystal packing shows a moderate density (1.175 g cm^−3^) among the cocrystals, which probably facilitate the water penetration into the crystal structure and its dissociation. In contrast, 4,4’-bipyridine is also doubly protonated; however, it exhibits the highest crystal density (1.206 g cm^−3^). The tighter crystal packing probably limits the water penetration into the crystal, leading to reduced apparent solubility and no dissociation of the cocrystal. In the case of tetramethylpyrazine, the coformer is only mono protonated, while the crystal packing density is the lowest (1.131 g cm^−1^). Despite the lowest crystal density, the reduced protonation probably contributes into similar dissolution behaviour comparable to CBN-BP.

Table SI 2. Intrinsic dissolution rate of the CBN and its cocrystals.

| Solid form | Average dissolution rate (μg min^−1^ cm^−2^) |
| --- | --- |
| CBN | 10.56 ± 0.95 |
| CBN-BP | 8.02 ± 0.68 |
| CBN-PI form III | 10.21 ± 0.72 |
| CBN-TMP 2:1 | 7.83 ± 0.20 |

a)

b)

c)

d)

Figure SI 14. Dissolution rate profiles of a) CBN, b) CBN-BP, c) CBN-PI form III
and d) CBN-TMP (2:1).

a)

b)

c)

d)

Figure SI 15. XRPD patterns after IDR measurement of a) CBN, b) CBN-BP,
c) CBN-PI form III and d) CBN-TMP 2:1. Colour of the star represents the origin of the peak.

# SI 3 Crystal Morphology and Calculated Interaction Energy of CBG and Its Cocrystals

## 3.1 CBG

The strongest calculated interaction (Figure SI 16a) between two molecules of CBG is −69.2 kJ mol^−1^. The predicted morphology (Figure SI 16b) shows that on the largest faces are hydrophobic groups (aliphatic tails) exposed, while on the smaller faces the combination of hydrophobic and hydrophilic (hydroxyl group) is exposed.

Figure SI 16. Crystal structure of CBG of a) calculated interaction energy,
b) predicted morphology.

## 3.2 CBG-PI

The strongest calculated interaction energy (−54.7 kJ mol^−1^, Figure SI 17a) occurs between the CBG and PI molecules and corresponds to the hydrogen-bonding direction. According to the predicted morphology (Figure SI 17b) aliphatic groups of CBG molecule are mostly exposed on the largest facets. The hydroxyl groups are exposed along these chains and are likely partially shielded by the surrounding hydrophobic moieties.

Figure SI 17. Crystal structure of CBG-PI of a) calculated interaction energy,
b) predicted morphology.

## 3.3 CBG-TMP form I

The strongest interaction (−47.9 kJ mol^−1^, Figure SI 18a) occurs between the molecules of TMP and CBG, which is involved in the hydrogen bond direction. The predicted morphology (Figure SI 18b) reveals that the largest crystal facets are exposing mostly hydrophobic (aliphatic chain) parts, which result in a surface overall more hydrophobic than hydrophilic.

Figure SI 18. Crystal structure of CBG-TMP form I of a) calculated interaction energy,
b) predicted morphology.

# SI 4 Dynamic Vapour Sorption

Figure SI 19. XRPD patterns after DVS measurement CBN-TMP (1:1) form I and II compared
to the pure form of CBN-TMP (1:1) form I and II and CBN-TMP 2:1.
Colour of the star represents the origin of the peak.

Table SI 3. Calculated energetic contributions to the particle energy
of CBN-TMP polymorphs.

|  | *E*_particle_  (kJ mol^−1^) | *E*_particle_ per unit cell (kJ mol^−1^) | *E*_latt_  (kJ mol^−1^) | Δ*E*_conf_  (kJ mol^−1^) | 0.5∑*x*_(hkl)_ *E*_(hkl)_ (kJ mol^−1^) |
| --- | --- | --- | --- | --- | --- |
| Form I | −111.1 | −444.3 | −120.9 | 1.42 | −8.5 |
| Form II | −106.4 | −425.6 | −118.2 | 4.34 | −7.5 |

b)

a)

Figure SI 20. Time-dependent XRPD patterns of a) CBN-TMP (1:1) form I
and b) CBN-TMP (1:1) form II.

# SI 5 Forced Degradation Study

## 5.1 Representative UPLC Chromatograms

### 5.1.1 CBD

CBD purity: 99.3 %

Figure SI 21. UPLC chromatogram of non-stressed CBD.

CBD purity: 98.9 %

Figure SI 22. UPLC chromatogram of CBD after thermal stressing (3 days, 80 °C, 75 % RH).

CBD purity: 99.1 %

Figure SI 23. UPLC chromatogram of CBD after photostability stressing.

CBD purity: 77.3 %

Figure SI 24. UPLC chromatogram of CBD after pH-dependent stressing (pH 6.7,
final sampling point).

CBD purity: 88.5 %

Figure SI 25. UPLC chromatogram of CBD after AIBN-induced oxidative stressing.

CBD purity: 97.8 %

Figure SI 26. UPLC chromatogram of CBD after peroxide-induced oxidative stressing.

### 5.1.2 CBN

CBN purity: 98.8 %

Figure SI 27. UPLC chromatogram of non-stressed CBN.

CBN purity: 98.1 %

Figure SI 28. UPLC chromatogram of CBN after thermal stressing (3 days, 80 °C, 75 % RH).

CBN purity: 98.8 %

Figure SI 29. UPLC chromatogram of CBN after photostability stressing.

CBN purity: 78.7 %

Figure SI 30. UPLC chromatogram of CBN after pH-dependent stressing (pH 6.7,
final sampling point).

CBN purity: 97.9 %

Figure SI 31. UPLC chromatogram of CBN after AIBN-induced oxidative stressing.

CBN purity: 98.8 %

Figure SI 32. UPLC chromatogram of CBN after peroxide-induced oxidative stressing.

### 5.1.3 CBG

CBG purity: 99.0 %

Figure SI 33. UPLC chromatogram of non-stressed CBG.

CBG purity: 96.9 %

Figure SI 34. UPLC chromatogram of CBG after thermal stressing (3 days, 80 °C, 75 % RH).

CBG purity: 98.5 %

Figure SI 35. UPLC chromatogram of CBG after photostability stressing.

CBG purity: 67.6 %

Figure SI 36. UPLC chromatogram of CBG after pH-dependent stressing (pH 6.7,
final sampling point).

CBG purity: 87.2 %

Figure SI 37. UPLC chromatogram of CBG after AIBN-induced oxidative stressing.

CBG purity: 97.9 %

Figure SI 38. UPLC chromatogram of CBG after peroxide-induced oxidative stressing.

## 5.2 XRPD Analysis of Stressed Samples

a)

b)

c)

d)

Figure SI 39. XRPD patterns after thermal stability of a) CBN, b) CBN-BP, c) CBN-PI form III and d) CBN-TMP 2:1 compared to the pure cocrystals and starting materials.

a)

b)

c)

Figure SI 40. XRPD patterns after thermal stability of a) CBG, b) CBG-PI and c) CBG-TMP form I compared to the pure cocrystals and starting materials.

a)

b)

c)

d)

e)

f)

Figure SI 41. XRPD patterns after irradiation of a) CBD, b) CBD-BP, c) CBD-CR, d) CBD-PI,
e) CBD-PR and f) CBD-TMP compared to the pure cocrystals and starting materials.

a)

b)

c)

d)

Figure SI 42. XRPD patterns after irradiation of a) CBN, b) CBN-BP, c) CBN-PI form III
and d) CBN-TMP 2:1 compared to the pure cocrystals and starting materials.

a)

b)

c)

Figure SI 43. XRPD patterns after irradiation of a) CBG, b) CBG-PI and c) CBG-TMP form I
compared to the pure cocrystals and starting materials.

# Literature

Uncategorized References

(1) Groom, C. R.; Bruno, I. J.; Lightfoot, M. P.; Ward, S. C. The Cambridge Structural Database. *Acta Cryst. B* **2016**, *72* (2), 171-179, doi: 10.1107/S2052520616003954.

(2) Bruno, I. J.; Cole, J. C.; Edgington, P. R.; Kessler, M.; Macrae, C. F.; McCabe, P.; Pearson, J.; Taylor, R. New software for searching the Cambridge Structural Database and visualizing crystal structures. *Acta Cryst. B* **2002**, *58* (3 Part 1), 389-397, doi: 10.1107/S0108768102003324.

(3) Mkrtchyan, G.; Hoerner, J. K.; Couch, R. W.; Bis, J. A.; Carino, S. A. R. Cocrystals of cannabinoids. US WO2021138610A1, 2023.

(4) Tesson, N.; Trilla Castano, M.; Comely, A. C. Solid compositions of cocrystals of cannabinoids. Spain WO2020089424A1, 2021.

(5) Koryťáková, A.; Chatziadi, A.; Rohlíček, J.; Zmeškalová, E.; Beránek, J.; Šoóš, M. Stability study and structural insights into cannabidiol cocrystals. *CrystEngComm* **2025**, *27* (14), 2154-2165, doi: 10.1039/D4CE01314J.

(6) Koryt’áková, A.; Chatziadi, A.; Rohlíček, J.; Zmeškalová, E.; Beránek, J.; Šoóš, M. Polymorphism in Cannabinol Piperazine Cocrystals: Structural, Morphological, and Energetic Perspectives. *Cryst. Growth Des.* **2025**, *25* (24), 10588-10599, doi: 10.1021/acs.cgd.5c01390.

(7) *Agilent CrysAlis PRO*; Agilent Technologies Ltd.: Yarnton, England, 2014. (accessed 02.01.2024).

(8) Betteridge, P. W.; Carruthers, J. R.; Cooper, R. I.; Prout, K.; Watkin, D. J. CRYSTALS version 12: software for guided crystal structure analysis. *J. Appl. Crystallogr.* **2003**, *36* (6), 1487, doi: 10.1107/S0021889803021800.

(9) Petříček, V.; Palatinus, L.; Plášil, J.; Dušek, M. Jana2020 – a new version of the crystallographic computing system Jana. *Z. Kristallogr. Cryst. Mater.* **2023**, *238* (7-8), 271-282, doi: 10.1515/zkri-2023-0005.

(10) Rohlíček, J.; Hušák, M. MCE2005–a new version of a program for fast interactive visualization of electron and similar density maps optimized for small molecules. *J. Appl. Cryst.* **2007**, *40* (3), 600-601, doi: 10.1107/S0021889807018894.

(11) Favre-Nicolin, V.; Cerny, R. FOX, `free objects for crystallography': a modular approach to ab initio structure determination from powder diffraction. *J. Appl. Crystallogr.* **2002**, *35* (6), 734-743, doi: 10.1107/S0021889802015236.

(12) Louër, D.; Boultif, A. Some further considerations in powder diffraction pattern indexing with the dichotomy method. *Powder Diffr.* **2014**, *29* (S2), S7-S12, doi: 10.1017/S0885715614000906.

(13) Manap, M. R. A. Experimental Crystal Structure Determination, CCDC 2217220. 2022.

(14) *ChemSketch*; Advanced Chemistry Development, Inc.: Toronto, Canada, 2022. www.acdlabs.com (accessed 19.8.2025).

(15) Mackenzie, C. F.; Spackman, P. R.; Jayatilaka, D.; Spackman, M. A. CrystalExplorer model energies and energy frameworks: extension to metal coordination compounds, organic salts, solvates and open-shell systems. *IUCrJ* **2017**, *4* (5), 575-587, doi: 10.1107/S205225251700848X.

(16) Ahuja, D.; Svärd, M.; Lusi, M.; Rasmuson, Å. C. Solution and calorimetric thermodynamic study of a new 1 : 1 sulfamethazine–3-methylsalicylic acid co-crystal. *CrystEngComm* **2020**, *22* (20), 3463-3473, doi: 10.1039/D0CE00498G.

(17) Chatziadi, A.; Skorepova, E.; Jirat, J.; Rohlíček, J.; Šoóš, M. Characterization and Insights into the Formation of New Multicomponent Solid Forms of Sofosbuvir. *Cryst. Growth Des.* **2022**, *22* (5), 3395-3404, doi: 10.1021/acs.cgd.2c00207.

(18) Gong, N.; Yu, H.; Wang, Y.; Xing, C.; Hu, K.; Du, G.; Lu, Y. Crystal Structures, Stability, and Solubility Evaluation of a 2:1 Diosgenin–Piperazine Cocrystal. *Nat. Prod. Bioprospect.* **2020**, *10* (4), 261-267, doi: 10.1007/s13659-020-00256-y.

(19) Pagano, J. M.; Goldberg, D. E.; Fernelius, W. C. A thermodynamic study of homopiperazine, piperazine and N-(2-aminoethyl)-piperazine and their complexes with copper (II) ion. *J. Phys. Chem.* **1961**, *65* (6), 1062-1064, doi: 10.1021/j100824a513.

Data availability

Crystallographic data for 2512838 (CBN-BP), 2512839 (CBN-TMP 1:1 form I), 2512840 (CBN-TMP 1:1 form II) and 2512841 (CBN-TMP 2:1)) has been deposited at the Cambridge Structural Database.
